# Supplementary material for: The methylomic landscape of human articular cartilage development contains epigenetic signatures of osteoarthritis risk
Source: Am J Hum Genet. 2024 Nov 22;111(12):2756–72. doi: 10.1016/j.ajhg.2024.10.017 (PMC11639090; doi:10.1016/j.ajhg.2024.10.017)
Supplement: Document S1. Figures S1–S10 and supplemental methods [file mmc1.pdf]

The American Journal of Human Genetics, Volume *111*

Supplemental information

The methylomic landscape of human articular cartilage development contains epigenetic signatures of osteoarthritis risk

Euan McDonnell, Sarah E. Orr, Matthew J. Barter, Danielle Rux, Abby Brumwell, Nicola Wrobel, Lee Murphy, Lynne M. Overman, Antony K. Sorial, David A. Young, Jamie Soul, and Sarah J. Rice

Supplemental Data

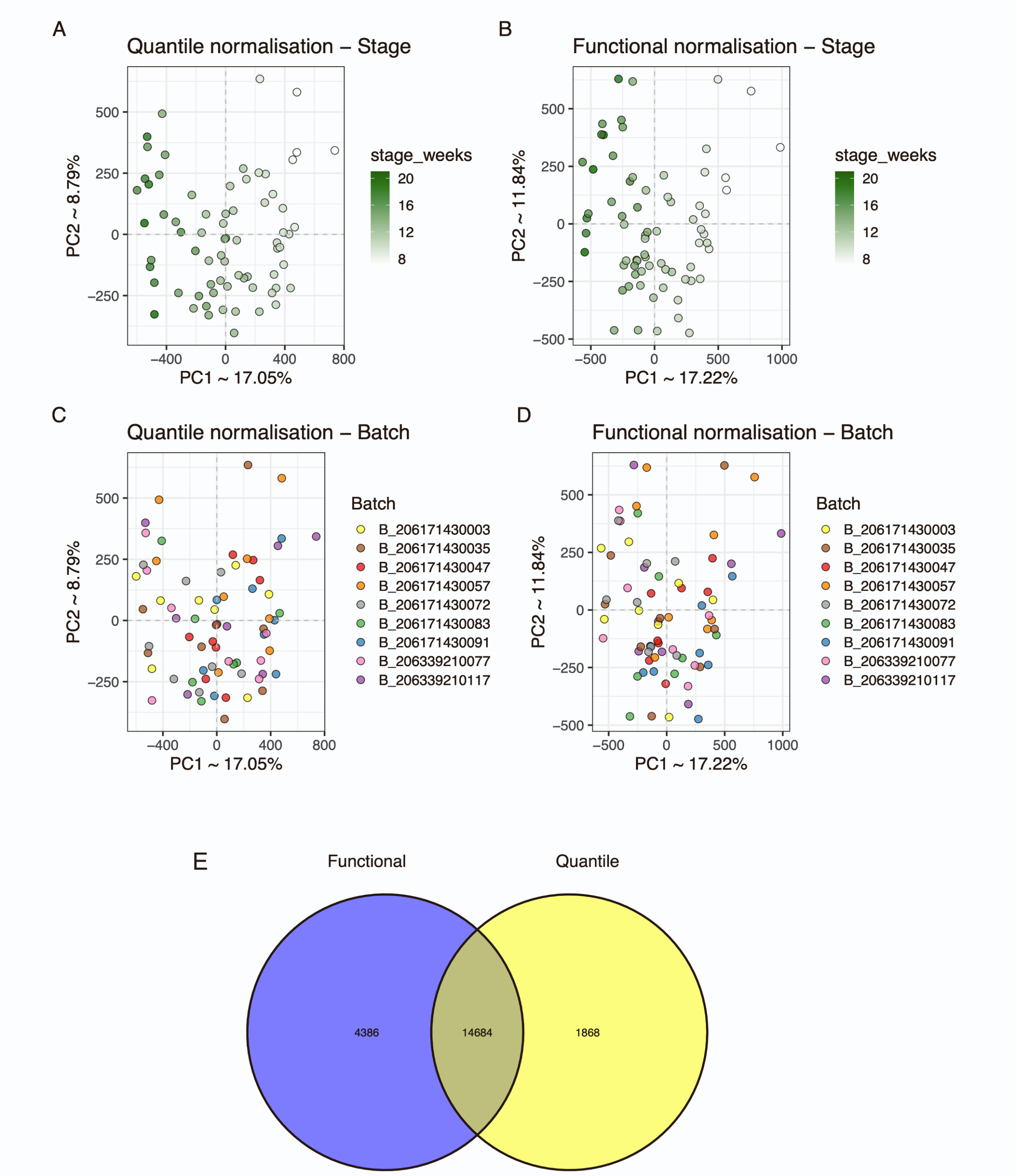

Supplemental Figure 1. Sensitivity of principal component analysis to normalisation approach. **A-D**, Principal component analysis of quantile (A, C) or functional (B, D) normalised methylation data coloured by sample developmental stage or Sentrix ID (batch) shows limited effect of normalisation choice. **E**, Venn diagram of the significant Bonferroni corrected developmental differential methylated probes shows high overlap of differentially methylated probes with quantile and functional normalisation.

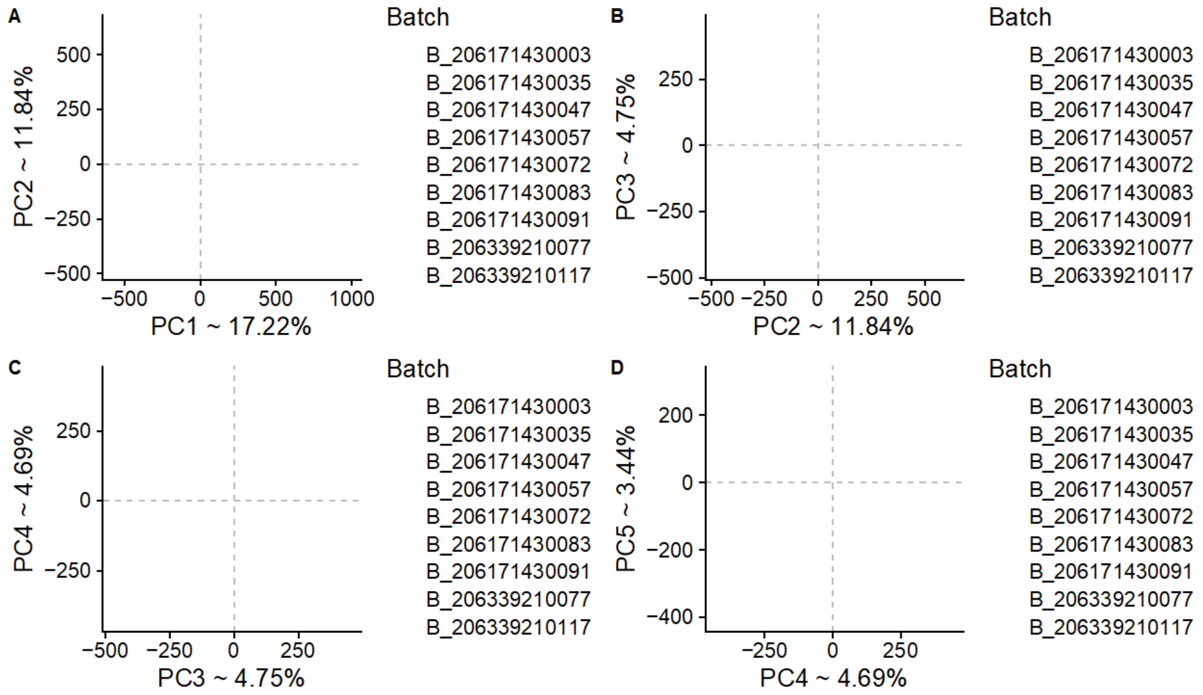

Supplemental Figure 2. Assessment of batch effects by principal component analysis of the 71 foetal cartilage samples used in this study. Principal component analysis of normalised DNAm samples coloured by Sentrix ID shows evidence of weak batch effects, observable in association with PC4.

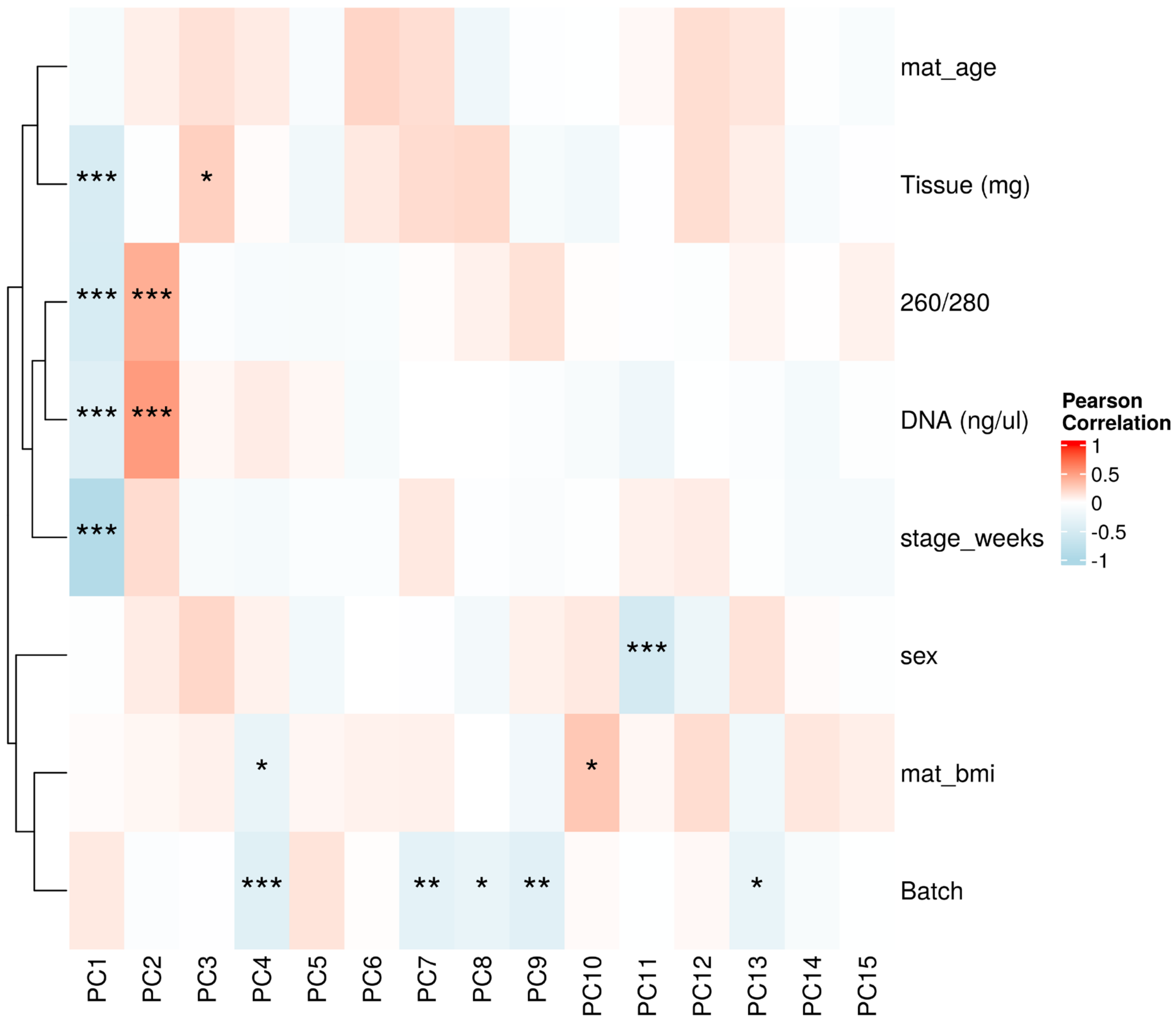

Supplemental Figure 3. Heatmap of Pearson's correlations between calculated methylation principal components and known biological and technical covariates. * p-value < 0.05, ** p-value < 0.01, *** p-value < 0.001

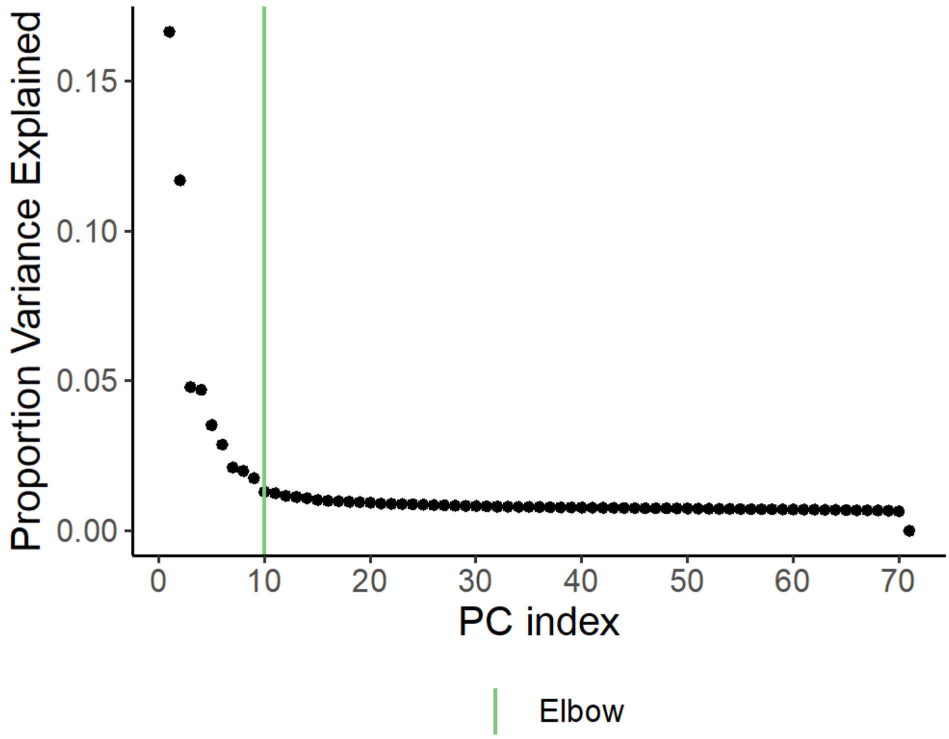

Supplemental Figure 4. Selection of methylation principal components for mQTL analysis. Principal component analysis was performed on the methylation M-values. The variance explained by each principal component is shown and 10 principal components were selected for inclusion in the mQTL analysis using the elbow method of PCAForQTL.

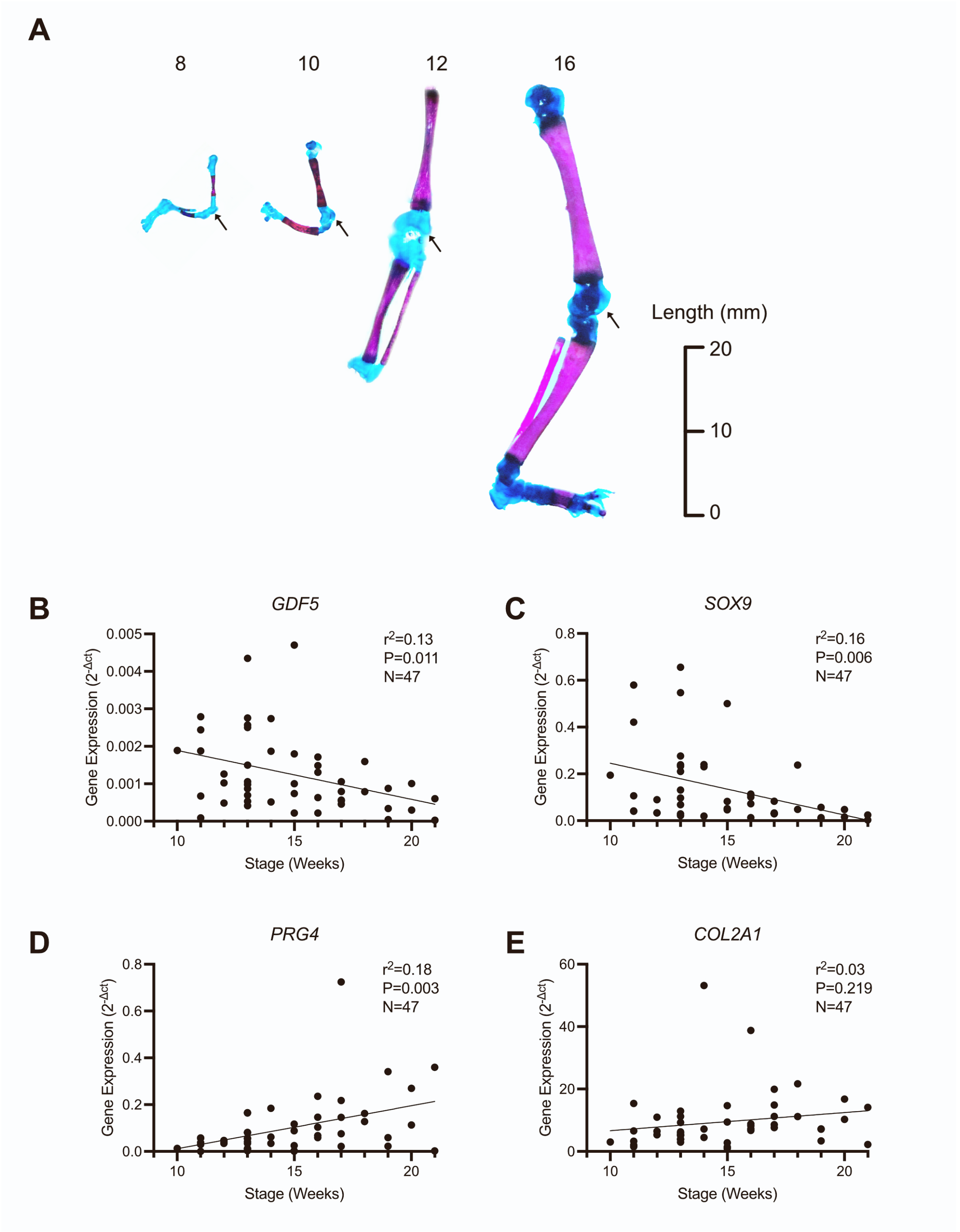

Supplemental Figure 5. Characterisation of the developing human knee cartilage samples. A, Alcian blue (cartilage) and alizarin red (bone) staining of human foetal femur at (L-R) 8, 10, 12, and 16 pcw. Arrows indicate the lower femoral articular surface **B-E,** Gene expression of cartilage progenitor (*GDF5* and *SOX9*) and chondrocyte (*PRG4* and *COL2A1*) makers in the samples. Simple linear regression was performed by the developmental stage.

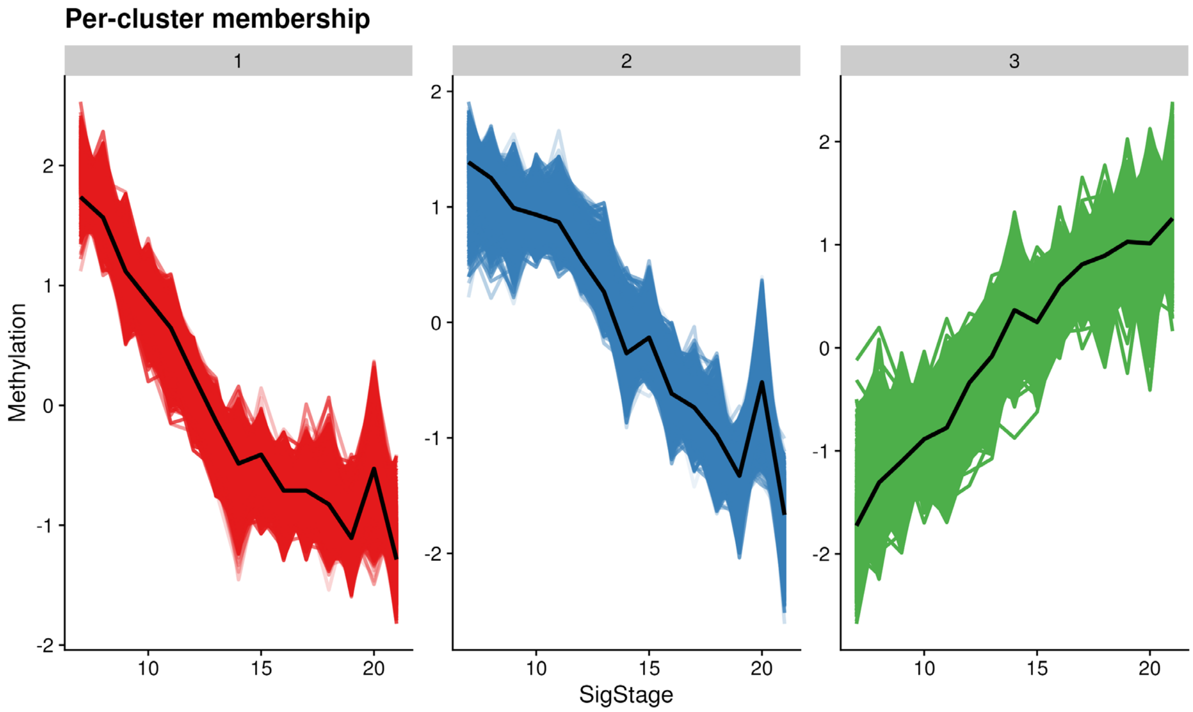

Supplemental Figure 6. The identified dDMPs fell into three distinct clusters based upon the methylation trends across the developmental window. The mfuzz plot for each cluster is displayed. Cluster 1, red; Cluster 2, blue; Cluster 3, green. Methylation is plotted using M-values.

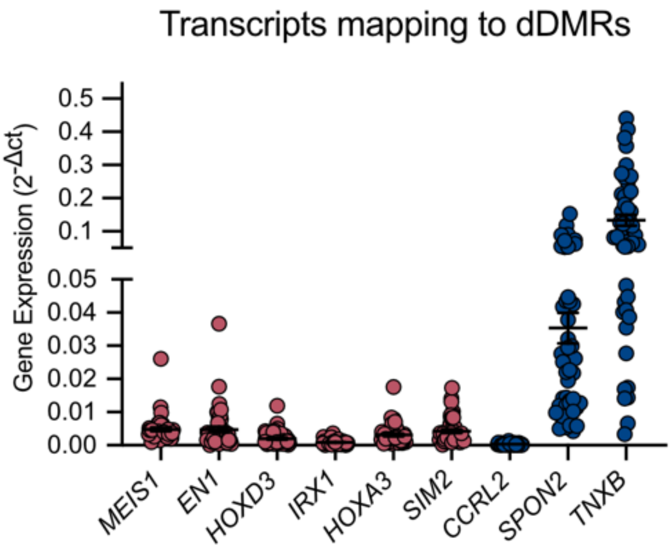

Supplemental Figure 7. Expression of genes mapping to the top hypermethylated (red) and hypomethylated (blue) developmental DMRs (dDMRs).

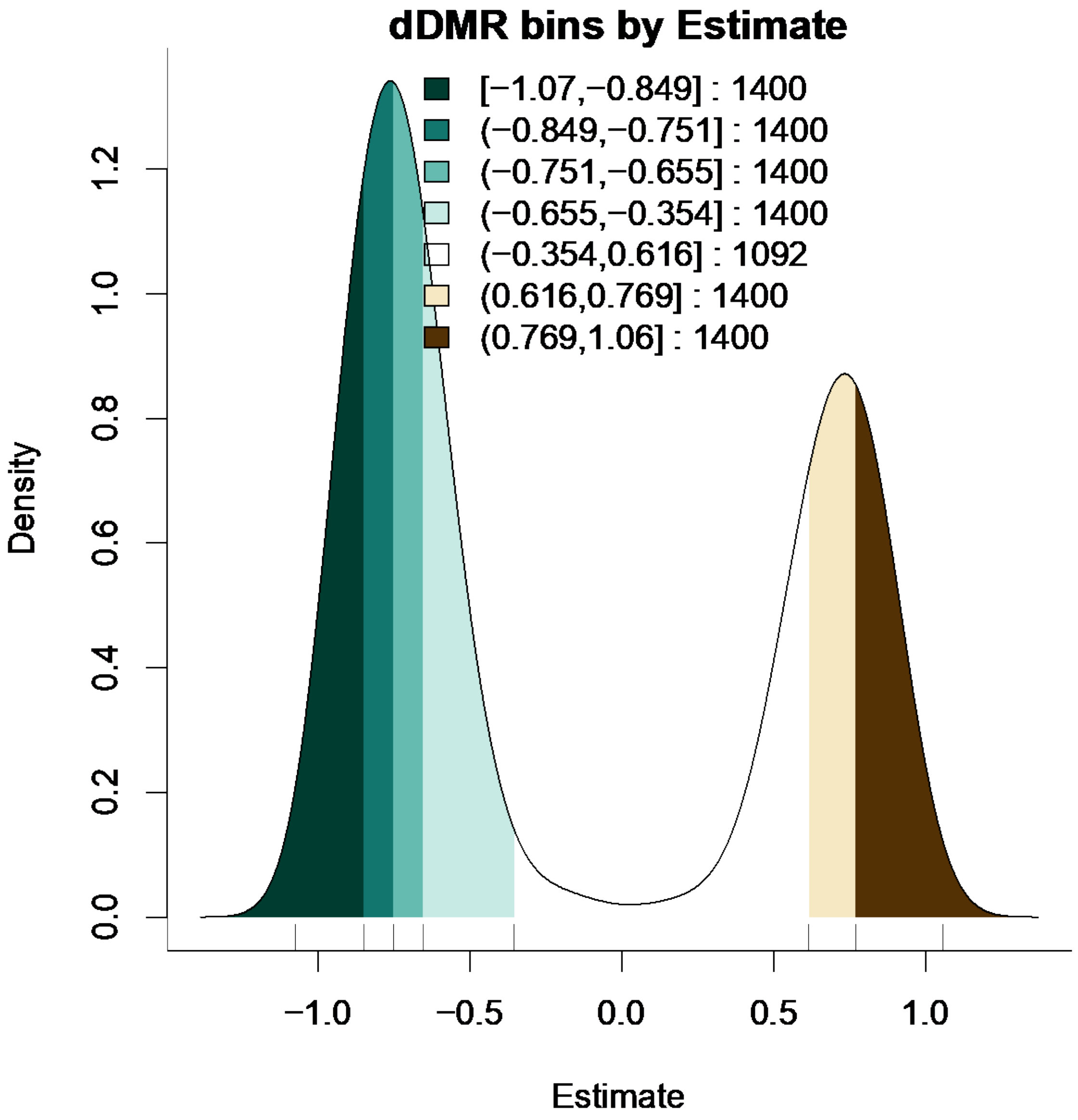

Supplemental Figure 8. Developmental differentially methylated region (dDMR) bins for transcription factor motif analysis. Histogram of the equally sized groups of developmental DMRs binned by Estimate (log2 fold change) that were used for transcription factor motif analysis with monaLisa.

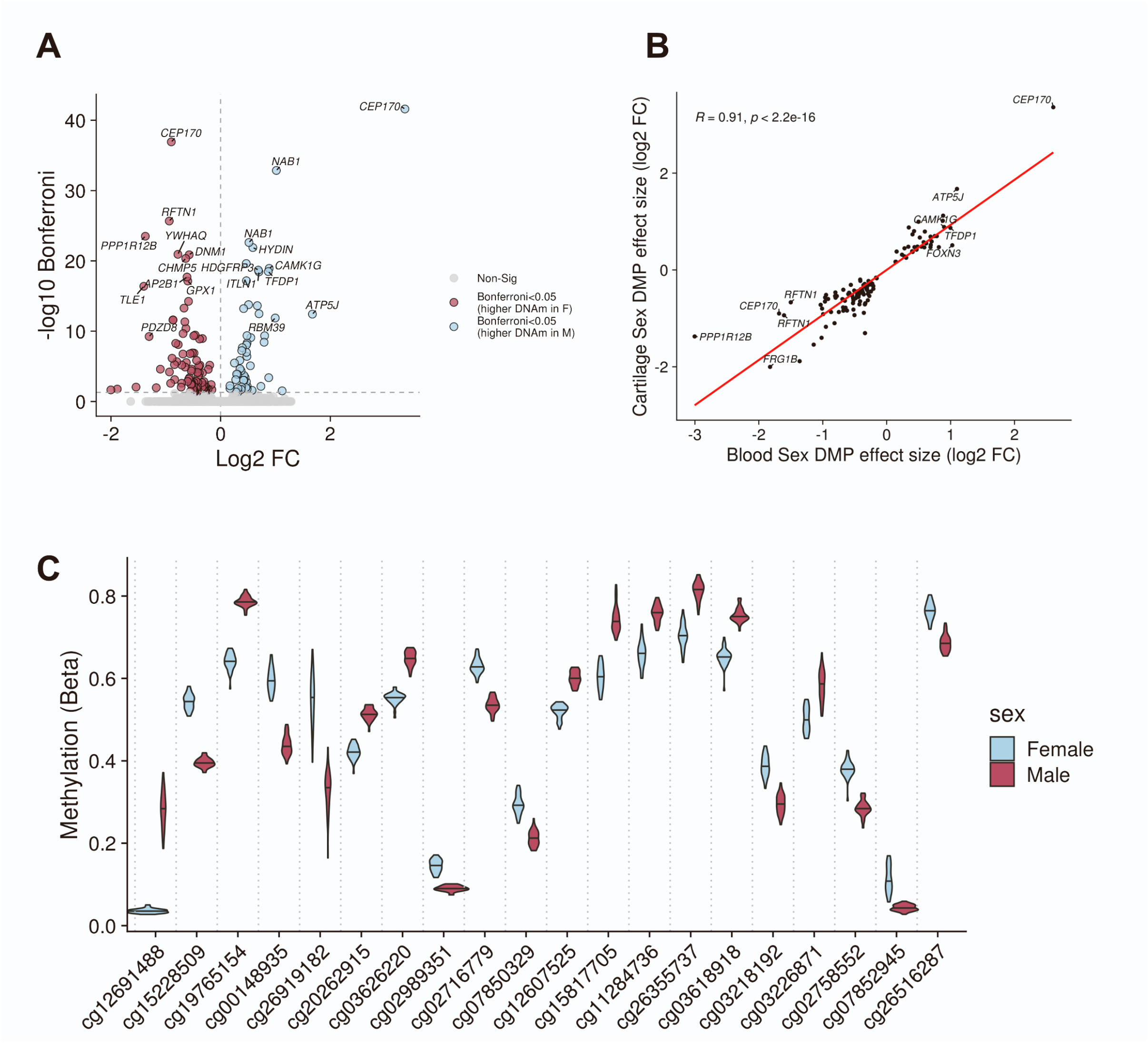

Supplemental Figure 9. Sexual dimorphism in DNA methylation within human developmental cartilage. A, Volcano plot of sDMPs. Grey, non-significant sDMPs (Bonferroni <0.05); Blue, significant sDMPs with higher methylation in male (M) samples (Bonferroni < 0.05); Red, significant sDMPs with higher methylation in female (F) samples (Bonferroni < 0.05); B, Comparison of effect sizes (log2 fold change) for overlapping significant (Bonferroni < 0.05) sDMPs in neonatal blood reported in Santos et al. and foetal cartilage. Genes nearest to the top hyper- and hypomethylated probes are highlighted; D, Violin plot of methylation beta values in human foetal cartilage samples at the most significant sDMP CpGs. Red, male; Blue, female.

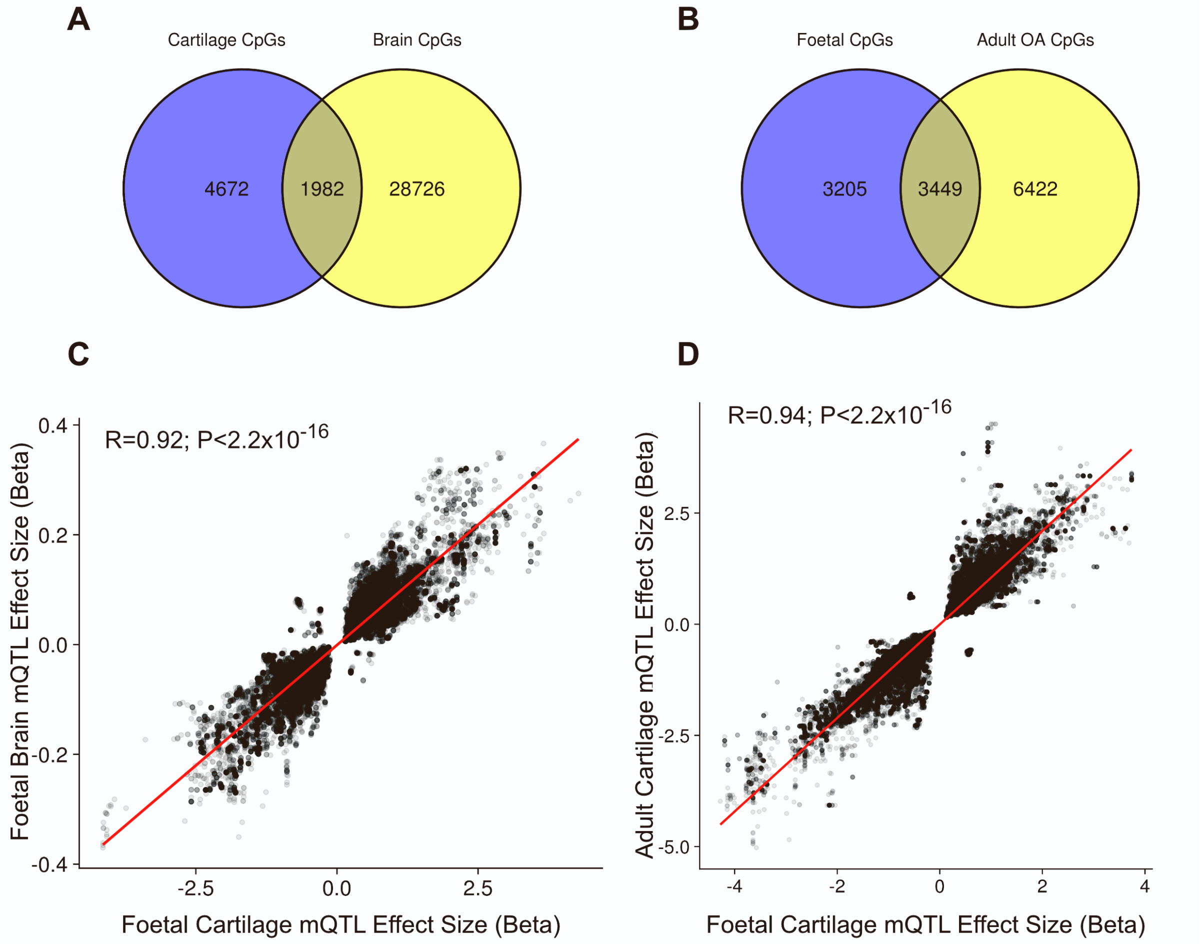

Supplemental Figure 10. Comparison of mQTLs with existing foetal brain and adult osteoarthritic cartilage datasets. A, Venn diagram of the overlapping significant CpGs involved in mQTLs in foetal brain and foetal cartilage mQTLs; B, Venn diagram of the overlapping significant CpGs involved in mQTLs in adult low grade (macroscopically intact) osteoarthritic knee cartilage and foetal cartilage mQTLs; C, Comparison of effect sizes (beta) between the shared significant mQTLs in foetal brain and foetal cartilage mQTLs; D, Comparison of effect sizes (beta) between the shared significant mQTLs in adult cartilage and foetal cartilage mQTLs**.**

Sample information

The developmental maturity of the embryonic samples (<10 gestation weeks) are staged according to the Carnegie staging classification system, which are determined using the HDBR staging guide (https://hdbratlas.org/staging-criteria/carnegie-staging.html) which itself is based upon the staging guide presented in Bullen and Wilson (1997)[1]. Fetal samples are staged from the time of conception, which is estimated by HDBR during sample collection (https://hdbratlas.org/staging-criteria/fetal-staging.html) based on the methods of Hern[2]. All of the data presented here considers the development of the embryo using Carnegie stage (CS) and fetus in post-conception weeks (pcw). Detailed information about the sample characteristics is included in Table S1, along with available maternal data.

Sample isolation and nucleic acid extraction

Nascent articular and epiphyseal cartilage tissue (20-30mg) was taken from the distal end of the developing femur. As anticipated, adipose tissue was visible within the joint from ~19pcw and was thoroughly removed before cartilage isolation. In all cases, tissue was homogenized in 2ml screw-cap tubes containing 600µl RTL lysis buffer (AllPrep, Qiagen) at 2400rpm for 2.5min in 2mm Zirconia beads using the Mini BeadBeater 24 (both from BioSpec Products, USA). Samples were centrifuged at 13,000rpm for 30s before further homogenizing at 2400rpm for 5min. Samples were centrifuged again before clarification through 0.2µM nylon filters (GeneFlow, UK). DNA was isolated using AllPrep (Qiagen) and RNA was isolated using Norgen DNA/RNA mini purification kit (Norgen BioTek Corp, Canada).

Staining of bone and cartilage tissues

Fetal samples were stained following a previously published protocol[3], adapted for human tissue. Briefly, excess tissue was removed from the limb before fixing in 70% ethanol for 24hrs, then 95% ethanol for 24hrs. Ethanol was replaced with 0.03% Alcian blue solution for 1-3 days, depending on fetal stage, then washed with 95% ethanol for 6 hours, before replacement with 2% KOH solution for 12-24 hours. The KOH was changed to 0.005% Alizarin red for 12-

24 hours, before clearance in 1%KOH:20% glycerol solution for 1-5 days. All stages took place with gentle agitation. The samples were imaged using brightfield optics.

DNA methylation quality control

The minfi[4] R package (v1.4.6) was used to read in, process and perform quality control checks on methylation microarray data[5]. Bisulfite conversion and sex prediction checks were performed, and one sample was excluded from the analysis due to a mismatch between predicted and labelled sex. Non-autosomal sites were then excluded. Next, undetected probes (sum of Beta values across all samples equal to 0) and probes detected in less than 3 replicate beads were removed. Probes were then filtered to retain those with a detection P-value <0.001 and any probes with missing data (marked as not applicable, NA) in any samples were excluded. Functional normalization was then applied to the microarray data. Probes covered by known single nucleotide variants (SNVs) and cross-reactive probes were removed using the maxprobes R package (v0.02)[5]. After quality control 678,267 probes representing CpG sites were retained for analysis.

Potential outlier probes were highlighted using the Tukey method implemented in the WateRMelon (v.2.4.0)[6] function pwod with default parameters. Outlier probes were flagged in the downstream DMP and mQTL results, but not removed or masked from analysis due to their observed association with developmental stage.

DNA methylation exploratory data analysis

Methylation data were transformed to M-values and filtered to remove the bottom 5% of probes by variance. Principal component analysis (PCA) was performed using the factoExtra R package (v1.0.7), using Z-scaled data as input (Figure S1). This PCA analysis is hereby referred to as the "methylation PCA" and associated principal components (PC) are "methylation PCs". Visual assessment of batch effect on these PCA plots using Sentrix ID

indicated methylation PC4 was associated with batch (Figure S2). Sentrix ID was therefore incorporated as a covariate in downstream statistical models. To formally determine if known biological or technical covariates were associated with variance in the methylation data, we correlated (Pearson's r) methylation PC1-15 with stage, maternal age, maternal BMI, and sex variables (biological variables) and tissue mass (mg), A260/A280 ratio, DNA concentration (ng/µl) and batch variables (technical variables) (Figure S3). To investigate the presence of any unknown technical factors, we performed n.sva using the Leek method with a model that included the known methylation array IDs. No significant surrogate variables were detected, indicating no major unknown sources of technical variation.

Differential methylation and differential methylated region analyses

Prior to statistical analyses, all data were transformed to M values. Probe-level differential methylation analysis was performed using the limma R package[7] (v3.55.5), using both the standard hypothesis testing method (null hypothesis of log2FC equal to 0) and the Testing Relative to A Threshold (treat) hypothesis testing approach (null hypothesis of log2FC equal to or less than 0.1)[8]. This treat method was taken forward for stage-associated differential methylation analysis, while the standard hypothesis testing method was used for sex-associated differential methylation. Limma linear models for methylation analysis included maternal BMI, maternal age and developmental stage as Z-scaled covariates, with sex as a binary factor. Coefficients and p-values were calculated with developmental stage and sex for developmental- and sex-associated CpGs, respectively. Reference-based analysis was not performed to adjust for variable cell populations in samples due to the homogeneity of cartilage tissue (single cell type, chondrocytes). Post-model fitting and testing, sex- and stage-DMPs were defined by filtration at a Bonferroni P-value of 0.05.

We investigated potential enrichment for up or down regulated DMPs in imprinted genes (obtained from geneimprint.com). We utilized gometh of the MissMethyl package[9] to test for

significant enrichment. No statistically significant enrichment was observed (P>0.05).

Differential methylated region (DMR) analysis with respect to the developmental stage was performed using the dmrff R package (v1.1.0) on the output p-values and standard errors of the probe-level differential methylation analysis. Probes were tested for association into a DMR with a maximum gap between probes of 1000 bp and DMRs defined as containing at least 2 probes and comprising the region between the 5' and 3'-most constituent probes. Resultant candidate sex DMRs (sDMRs) and developmental DMRs (dDMRs) were filtered by an Bonferroni of 0.05.

Sensitivity to Normalization Approach

To examine the sensitivity of the DMP analysis to the choice of methylation normalization, we also performed both quantile and functional normalization. The resulting PCAs showed close similarity after both modes of normalization and high overlap of differentially methylated probes (Figure S1). Functional normalization, that uses control probes as part of the normalization, was selected for use throughout the main analysis due to a higher percentage variance explained by PC2 and to minimize the potential for any confounding technical variables.

DMP clustering with mFuzz

M values for significant stage DMPs were clustered using the Mfuzz R package[10] (v2.62.0) with the optimal cluster number chosen as 3, based on the elbow method. Minimum cluster membership was chosen as 0.99 with a seed value of 5 and the "fuzzifier" parameter was calculated as 1.15 using the "mestimate" function of Mfuzz.

DMR overlap analysis

DMR regions were overlapped within the bounds of Assay for Transposase Accessible Chromatin (ATAC)-Seq peaks previously generated in 12pcw foetal knee (distal femoral)

cartilage[11] and the 15 chromatin-state model predictions for human cultured chondrocytes (E049) downloaded from the NIH ROADMAP Epigenomics Mapping Consortium[12]. Prior to overlap, the 15 ROADMAP states were collapsed into 5 functional classifications: enhancer (Enh, EnhG, EnhBiv), repressed (ZNF/Rpts, Quies, Het, ReprPC, ReprPCWk), transcription start site (TssBiv, TssA), flanking transcription start site (TssAFlnk, BivFlnk) and transcribed (TxWk, Tx, TxFlnk). Regions were overlapped using the intersect function in the bedtools (v2.31.1) package[13]. Presented frequencies and proportions represent overlaps between DMRs and ATAC regions/ROADMAP state classes, not the frequencies of unique members of either of these features. State enrichments were performed using two-sided Fisher's exact tests, testing for frequencies of overlaps with hypo- or hypermethylated DMRs.

GO Meth term enrichment analysis.

Gene ontology (GO) term enrichment analysis was performed on MFuzz clusters and sex DMPs using the gometh of the MissMethyl package[9] (v1.36.0), while DMR enrichment analysis made use of the goregion function. The Gene Ontology (GO) database was used with array.type="EPIC" argument provided to provide DMP-gene annotations. All 678,267 CpGs detected above thresholds were provided as background for all comparisons. Terms were called as significant with an FDR threshold < 0.05.

TF Motif Enrichment Analysis

TF Motif PWMs were downloaded from HOCOMOCO v12[14] and filtered to retain motifs with a quality grade of C and above. MonaLisa (v1.9.0) was used to group all identified dDMRs into bins of 1400 DMRs by fold change[15]. To allow comparison between bins, the DMR sequences were trimmed to the median DMR size (253bp), centered around the center of each DMR (Figure S9). Enrichment statistics (Fisher's exact test) were calculated by comparing occurrences of each motif in a bin to its occurrences in all other bins. Hierarchical clustering of the motif similarity of the enriched motifs was used to visualize the enrichment scores.

Genotype calling and genotype data filtration and quality control

Genotypes were called from raw idat files using gencall and associated array cluster and manifest files. The resultant gtc files were converted to vcf via the gtc_to_vcf.py script of the GTCtoVCF codebase (https://github.com/Illumina/GTCtoVCF). PLINK (v1.90b6.21) was used for subsequent analytic steps, except where otherwise stated[16]. Strand-flipped SNVs were identified by performing sample-wise merges with all remaining samples before all sample data was merged to one PLINK dataset. Genotype data filtering was performed using a minor allele frequency (MAF) > 0.01, while >0.975 was used as the variant and individual genotyping rate filtration threshold. X chromosome pseudoautosomal regions (PARs) were split to a separate chromosome, “26”. Sex and interrelatedness checks were performed using PLINK.

Imputation

Pre-imputation checks made use of the checkVCF utility (https://github.com/zhanxw/checkVCF/tree/master). All samples were submitted for imputation, while retaining only autosomes within each sample. Imputation was performed via submission to the Michigan Imputation Server (https://imputationserver.sph.umich.edu/) with population set to “EUR”, genome build as “hg19” and using the haplotype reference consortium (HRC) as reference population[17,18]. Imputed vcf files were then filtered to split multiallelic sites into separate records and sites filtered by $r^2>0.3$. Imputed variants were then filtered with MAF > 0.05, alongside >0.975 for both variant and individual genotyping rates. Pre-imputation and post-imputation processing made use of bcftools (v1.16) and post-imputation checks utilised the ic utility (v1.0.9) of the batch McCarthy Group Tools suite[19]. After imputation and filtering, 5,394,299 variants were used in the subsequent mQTL analysis.

1000 genomes project PCA

PLINK2 format data (pgen, pvar and psam files) for the 1000 genomes project were converted to PLINK format data (.bed, .bim and .fam) and these were used as the reference population data[20]. For both reference and study data, A-T and C-G alleles were excluded and multiallelic

sites were entirely excluded, using the “--biallelic strict” option. Next, sites of high linkage-disequilibrium (LD) were removed from the study data using a database of sites provided by the R package plinkQC (v0.3.4). Sites in the study data were then filtered with a 50kb sliding window, moving in 5kb steps and removing sites with a pairwise $r^2>0.2$. Sites in the reference data were then filtered by this pruned list of study data variants. Mismatched chromosome and position IDs were then identified and rectified, and variants orientated on the opposite strands between reference and study data were flipped, followed by the merging of the study and reference datasets. PCA was then performed using PLINK to obtain ancestry related principal components for inclusion in mQTL analysis.

Comparison to mQTL datasets

Fetal brain and adult low grade cartilage mQTLs were downloaded from http://epigenetics.essex.ac.uk/mQTL//All_Imputed_BonfSignificant_mQTLs.csv.gz and https://personal.broadinstitute.org/ryank/Kreitmaier_2022_mqtl_lgcart.txt.gz. For comparison between datasets the Bonferroni significant mQTLs and those CpGs present on the Illumina 450K array were retained. For effect size comparison mQTLs were normalized to the same reference allele and Pearson correlation performed.

Supplemental References

1. Strachan, T., Lindsay, S. (Susan), and Wilson, D.I. (David I.) (1997). Molecular genetics of early human development. 265.
2. Hern, W.M. (1984). Correlation of fetal age and measurements between 10 and 26 weeks of gestation. Obstetrics and Gynecology *63*, 26–32.
3. Rigueur, D., and Lyons, K.M. (2014). Whole-mount skeletal staining. Methods Mol Biol *1130*, 113–121. https://doi.org/10.1007/978-1-62703-989-5_9.
4. Aryee, M.J., Jaffe, A.E., Corrada-Bravo, H., Ladd-Acosta, C., Feinberg, A.P., Hansen, K.D., and Irizarry, R.A. (2014). Minfi: a flexible and comprehensive Bioconductor package for the analysis of Infinium DNA methylation microarrays. Bioinformatics *30*, 1363–1369. https://doi.org/10.1093/BIOINFORMATICS/BTU049.
5. Aryee, M.J., Jaffe, A.E., Corrada-Bravo, H., Ladd-Acosta, C., Feinberg, A.P., Hansen, K.D., and Irizarry, R.A. (2014). Minfi: a flexible and comprehensive Bioconductor package for the analysis of Infinium DNA methylation

microarrays. Bioinformatics *30*, 1363–1369. https://doi.org/10.1093/BIOINFORMATICS/BTU049.
6. Pidsley, R., Y Wong, C.C., Volta, M., Lunnon, K., Mill, J., and Schalkwyk, L.C. (2013). A data-driven approach to preprocessing Illumina 450K methylation array data. BMC Genomics *14*, 1–10. https://doi.org/10.1186/1471-2164-14-293/TABLES/2.
7. Ritchie, M.E., Phipson, B., Wu, D., Hu, Y., Law, C.W., Shi, W., and Smyth, G.K. (2015). limma powers differential expression analyses for RNA-sequencing and microarray studies. Nucleic Acids Res *43*, e47. https://doi.org/10.1093/NAR/GKV007.
8. Ritchie, M.E., Phipson, B., Wu, D., Hu, Y., Law, C.W., Shi, W., and Smyth, G.K. (2015). limma powers differential expression analyses for RNA-sequencing and microarray studies. Nucleic Acids Res *43*, e47–e47. https://doi.org/10.1093/NAR/GKV007.
9. Phipson, B., Maksimovic, J., and Oshlack, A. (2016). missMethyl: an R package for analyzing data from Illumina's HumanMethylation450 platform. Bioinformatics *32*, 286–288. https://doi.org/10.1093/BIOINFORMATICS/BTV560.
10. Kumar, L., and Futschik, M.E. (2007). Mfuzz: a software package for soft clustering of microarray data. Bioinformation *2*, 5–7. https://doi.org/10.6026/97320630002005.
11. Rice, S.J., Brumwell, A., Falk, J., Kehayova, Y.S., Casement, J., Parker, E., Hofer, I.M.J., Shepherd, C., and Loughlin, J. (2022). Genetic risk of osteoarthritis operates during human skeletogenesis. Hum Mol Genet. https://doi.org/10.1093/HMG/DDAC251.
12. Roadmap Epigenomics Consortium, Kundaje, A., Meuleman, W., Ernst, J., Bilenky, M., Yen, A., Heravi-Moussavi, A., Kheradpour, P., Zhang, Z., Wang, J., et al. (2015). Integrative analysis of 111 reference human epigenomes. Nature *518*, 317–329. https://doi.org/10.1038/nature14248.
13. Quinlan, A.R., and Hall, I.M. (2010). BEDTools: a flexible suite of utilities for comparing genomic features. Bioinformatics *26*, 841–842. https://doi.org/10.1093/BIOINFORMATICS/BTQ033.
14. Vorontsov, I.E., Eliseeva, I.A., Zinkevich, A., Nikonov, M., Abramov, S., Boytsov, A., Kamenets, V., Kasianova, A., Kolmykov, S., Yevshin, I.S., et al. (2024). HOCOMOCO in 2024: a rebuild of the curated collection of binding models for human and mouse transcription factors. Nucleic Acids Res *52*, D154–D163. https://doi.org/10.1093/NAR/GKAD1077.
15. Machlab, D., Burger, L., Soneson, C., Rijli, F.M., Schübeler, D., and Stadler, M.B. (2022). monaLisa: an R/Bioconductor package for identifying regulatory motifs. Bioinformatics *38*, 2624–2625. https://doi.org/10.1093/BIOINFORMATICS/BTAC102.
16. Purcell, S., Neale, B., Todd-Brown, K., Thomas, L., Ferreira, M.A.R., Bender, D., Maller, J., Sklar, P., De Bakker, P.I.W., Daly, M.J., et al. (2007). PLINK: a tool set for whole-genome association and population-based linkage analyses. Am J Hum Genet *81*, 559–575. https://doi.org/10.1086/519795.
17. Das, S., Forer, L., Schönherr, S., Sidore, C., Locke, A.E., Kwong, A., Vrieze, S.I., Chew, E.Y., Levy, S., McGue, M., et al. (2016). Next-generation genotype imputation service and methods. Nat Genet *48*, 1284–1287. https://doi.org/10.1038/NG.3656.

18. McCarthy, S., Das, S., Kretzschmar, W., Delaneau, O., Wood, A.R., Teumer, A., Kang, H.M., Fuchsberger, C., Danecek, P., Sharp, K., et al. (2016). A reference panel of 64,976 haplotypes for genotype imputation. Nat Genet *48*, 1279–1283. https://doi.org/10.1038/NG.3643.
19. Li, H. (2011). A statistical framework for SNP calling, mutation discovery, association mapping and population genetical parameter estimation from sequencing data. Bioinformatics *27*, 2987–2993. https://doi.org/10.1093/BIOINFORMATICS/BTR509.
20. Chang, C.C., Chow, C.C., Tellier, L.C.A.M., Vattikuti, S., Purcell, S.M., and Lee, J.J. (2015). Second-generation PLINK: rising to the challenge of larger and richer datasets. Gigascience *4*. https://doi.org/10.1186/S13742-015-0047-8.
