# Supplementary material for: The methylomic landscape of human articular cartilage development contains epigenetic signatures of osteoarthritis risk
Source: Am J Hum Genet. 2024 Nov 22;111(12):2756–72. doi: 10.1016/j.ajhg.2024.10.017 (PMC11639090; doi:10.1016/j.ajhg.2024.10.017)
Supplement: Document S2. Article plus supplemental information [file mmc3.pdf]

The methylomic landscape of human articular cartilage development contains epigenetic signatures of osteoarthritis risk

Graphical abstract

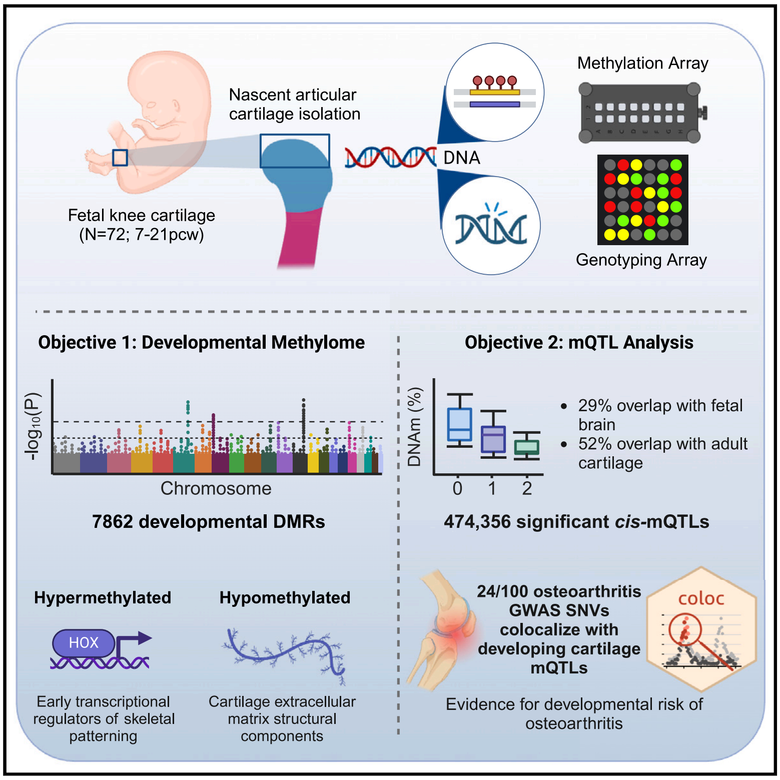

Authors

Euan McDonnell, Sarah E. Orr, Matthew J. Barter, ..., David A. Young, Jamie Soul, Sarah J. Rice

Correspondence

jamie.soul@liverpool.ac.uk (J.S.), sarah.rice@ncl.ac.uk (S.J.R.)

Emerging evidence supports developmental factors impacting osteoarthritis risk in older age. Here, we quantify the methylome of 72 human fetal knee cartilage samples and identify 24 independent osteoarthritis genetic risk SNVs colocalizing with developmental methylation quantitative trait loci. We report a subset of osteoarthritis epigenetic signatures active during skeletogenesis.

McDonnell et al., 2024, The American Journal of Human Genetics *111*, 2756–2772
December 5, 2024 © 2024 The Author(s). Published by Elsevier Inc. on behalf of American Society of Human Genetics.
https://doi.org/10.1016/j.ajhg.2024.10.017

ARTICLE

The methylomic landscape of human articular cartilage development contains epigenetic signatures of osteoarthritis risk

Euan McDonnell,[1] Sarah E. Orr,[2] Matthew J. Barter,[2] Danielle Rux,[3] Abby Brumwell,[2] Nicola Wrobel,[4] Lee Murphy,[4] Lynne M. Overman,[5] Antony K. Sorial,[2] David A. Young,[2] Jamie Soul,[6,7,*] and Sarah J. Rice[2,7,*]

Summary

Increasing evidence is emerging to link age-associated complex musculoskeletal diseases, including osteoarthritis (OA), to developmental factors. Multiple studies have shown a functional role for DNA methylation in the genetic mechanisms of OA risk using articular cartilage samples taken from aged individuals, yet knowledge of temporal changes to the methylome during human cartilage development is limited. We quantified DNA methylation at ~700,000 individual CpGs across the epigenome of developing human chondrocytes in 72 samples ranging from 7 to 21 post-conception weeks. We identified significant changes in 3% of all CpGs and >8,200 developmental differentially methylated regions. We further identified 24 loci at which OA genetic variants colocalize with methylation quantitative trait loci. Through integrating developmental and mature human chondrocyte datasets, we find evidence for functional effects exerted solely in development or throughout the life course. This will have profound impacts on future approaches to translating genetic pathways for therapeutic intervention.

Introduction

The development of human limbs and synovial joints commences between 4 and 8 post-conception weeks (pcw). During limb-bud outgrowth, mesenchymal condensations form the cartilage anlagen (driven by the transcriptional master-regulator, SOX9) to establish the rudimentary embryonic skeletal elements. The limb synovial joints are established through coordinated dedifferentiation of the nascent cartilage anlagen at the presumptive joint locations to form a unique pool of progenitors at each location known as the interzone. From these progenitors, all structures of the mature synovial joint are established, including articular cartilage, which contains only one cell type, the chondrocyte. For the knee joint, chondrogenesis within the femoral condyles is advanced by the start of 7 pcw, and a clear interzone has formed between the femur and the tibia, with full cavitation of the joint and formation of the synovial cavity commencing at 8 pcw.[1] This dynamic process of limb and articulating joint development requires the orchestrated expression gene transcripts essential for the development of defined and differentiated tissues.[2] The transcriptome is primarily regulated through the spatiotemporal expression of transcription factors (TFs), yet epigenetic processes, including DNA methylation (DNAm), both underlie and reinforce transitional plasticity during development.[3]

To date, DNAm remains the most extensively studied mammalian epigenetic mechanism. Methylation of DNA occurs at cytosine-phosphate-guanine (CpG) dinucleotides, of which there are approximately 28 million within the human genome. CpGs are recognized by DNA methyltransferase (DNMT) enzymes, which actively catalyze the addition of a methyl group from an *S*-adenosyl-L-methionine (SAM) donor to form 5-methylcytosine (5mC).[4] DNAm is intrinsically linked to transcriptional regulation, ostensibly to gene repression, by preventing binding of transcriptional activators to promoter regions and through the recruitment of repressive methyl-binding proteins. However, the relationship between DNAm and gene expression is far from straightforward, with gene body methylation often being associated with active transcription.[5] It is generally considered that DNAm within *cis*-regulatory elements (CREs) is repressive to the expression of the gene target.[6,7]

The methylome of human articular chondrocytes has been extensively studied, primarily in the context of the chronic joint disease osteoarthritis (OA). OA is a leading cause of disability among older adults globally, hallmarked by the degradation of articular cartilage in the joints, most commonly the hip or knee. Cartilage is an avascular and anural tissue, consisting of a single cell type, the chondrocyte, which is encased in a dense extracellular matrix. Studies of the aged chondrocyte methylome have revealed

[1]Computational Biology Facility, University of Liverpool, MerseyBio, Crown Street, Liverpool, UK; [2]Biosciences Institute, Newcastle University, Central Parkway, Newcastle upon Tyne, UK; [3]Orthopedic Surgery, UConn Health, Farmington, CT, USA; [4]Edinburgh Clinical Research Facility, University of Edinburgh, Edinburgh, UK; [5]Human Developmental Biology Resource, Newcastle University, International Centre for Life, Central Parkway, Newcastle upon Tyne, UK; [6]Institute of Systems, Molecular and Integrative Biology, University of Liverpool, Liverpool, UK
[7]These authors contributed equally
*Correspondence: jamie.soul@liverpool.ac.uk (J.S.), sarah.rice@ncl.ac.uk (S.J.R.)
https://doi.org/10.1016/j.ajhg.2024.10.017.
© 2024 The Author(s). Published by Elsevier Inc. on behalf of American Society of Human Genetics.
This is an open access article under the CC BY license (http://creativecommons.org/licenses/by/4.0/).

distinct epigenomic signatures between disease states[8,9] and between affected sites,[10,11] increasing our understanding of the joint specificity of disease. OA is multifactorial, with genetic risk factors contributing to ~30% of the lifetime risk of developing knee OA. The integration of DNAm data into genetic studies, such as the statistical fine mapping of genome-wide association studies (GWASs), has identified methylation quantitative trait loci (mQTLs) which colocalize with OA genetic risk signals.[8,11–13] This interplay between DNA sequence and CpG methylation status[14] has further been shown to underpin tissue-specific molecular mechanisms of gene expression within the joint.[15,16]

Developmental factors can also play a role in the risk of OA onset and progression in older age.[17] This includes both the shape of the joint[18–20] (which impacts the biomechanical properties and weight-bearing capacity) and the biochemical composition of the articular cartilage (which affects the resilience of the tissue to withstand stresses throughout the life course). Our recent targeted study of 39 CpGs investigated the presence of OA mQTLs in human fetal limbs at seven well-characterized OA genetic risk loci.[21] We identified that at 85% of the CpGs the significant OA-mQTLs replicated in the fetal tissues, demonstrating that functional epigenetic mechanisms associated with a musculoskeletal disease of older age can operate from the start of life.[21]

Murine knockout or inactivation of the three known DNMTs is lethal embryonically (*Dnmt1* and *Dnmt3b*) or postnatally (*Dnmt3a*), demonstrating the vital role of DNAm throughout development.[22,23] DNA-methylating enzymes are expressed in proliferating chondrocytes during embryonic development and persist in articular chondrocytes during postnatal development.[24] Investigation of the methylome of human developmental cartilage has been limited to two studies to date, which utilized small sample numbers ($n = 8$) falling within a narrow developmental window (14–19 pcw),[25] or, in the case of our previous study, applying targeted approaches that captured only a handful of CpGs.[21] Here, we present a comprehensive study of the methylomic landscape of human articular cartilage in a broader window of development to capture previously uninvestigated OA risk loci.

In this study, we had two primary objectives. First, we aimed to examine the DNA methylome of 72 fetal cartilage samples from the developing knee (distal femur) between 7 and 21 pcw. We identified the presence of differentially methylated regions across this developmental time frame and examine their enrichment in CREs and TF binding sites. Additionally, we investigated sexual dimorphism within the cartilage methylome during human development. Second, we tested the presence of developmental epigenomic changes contributing to the risk of OA in older life through the identification of fetal cartilage mQTLs colocalizing with OA genetic risk signals.

Methods

Sample collection

Cartilage tissue from the distal end of the developing human femur was supplied by the Medical Research Council (MRC)- and Wellcome Trust-funded Human Developmental Biology Resource (HDBR) at Newcastle University (http://www.hdbr.org, project number 200363), Newcastle upon Tyne. Tissues were obtained with appropriate maternal written consent and approval from the Newcastle and North Tyneside NHS Health Authority Joint Ethics Committee. HDBR is regulated by the UK Human Tissue Authority (HTA; www.hta.gov.uk) and operates by the relevant HTA Codes of Practice. Tissue for the HDBR is donated voluntarily by individuals who have had an elective termination of pregnancy from collaborating UK clinics. Further details of the sample source are outlined in the supplemental information. We isolated articular cartilage from the distal femur of 72 samples with developmental stages ranging from 7 to 21 pcw. The mean (±SEM) gestational stage was 13.3 ± 0.4 pcw (Figure 1A).

Generation of genotype and methylation data

DNA was genotyped using Illumina Global Screening Array v.3 BeadChip at the Edinburgh Clinical Research Facility. Bisulfite conversion of DNA (500 ng) was conducted using the EZ DNA methylation kit (Zymo) with Illumina-advised adjustments to the standard protocol. We profiled DNAm in the 72 samples using Illumina HumanMethylation850 EPIC v.1 microarrays via the Illumina iScan platform. Detected and autosomal probes were retained while those detected by fewer than three beads, with low detection p values ($p < 0.001$), present at known SNP sites, or known to be cross-reactive, were removed from the analysis.[26–28] One sample was dropped due to mismatch between predicted and labeled sex. The remaining 71 samples (full details of all samples used are presented in Table S1) had a minimum bisulfite conversion rate of 89.69%, and following quality control (QC) we retained data at 678,267 CpGs. Data were normalized via quantile or functional normalization using minfi[28] followed by exploratory data analysis with principal-component analysis (methylation PCA). Functional normalization was selected for use throughout the analysis (Figure S1). Developmental stage was found to strongly correlate with methylation principal component 1 (PC1), which accounted for 17% of the total variance in global methylation, and known experimental batch significantly correlated with PC4 (Figures 1B, S2, and S3).

Differential methylation and differential methylated region analyses

Following QC of the methylation data, exploratory data analysis of the methylation M values with PCA was performed to identify variables for inclusion in modeling (supplemental methods). Statistical modeling with the limma R package (v.3.55.5) was performed on the methylation M values to identify differentially methylated probes (DMPs) with the outcomes of interest—developmental stage (dDMPs) and sex (sDMPs)—while accounting for experimental batch, maternal body mass index (BMI), maternal age, sample tissue weight, DNA concentration, and DNA 260/280 ratio (Figure S3). No significant surrogate variables were identified with the SVA R package (v.3.46.0) after accounting for experimental batch. Probes with Bonferroni-adjusted p values of <0.05 were considered differentially methylated.

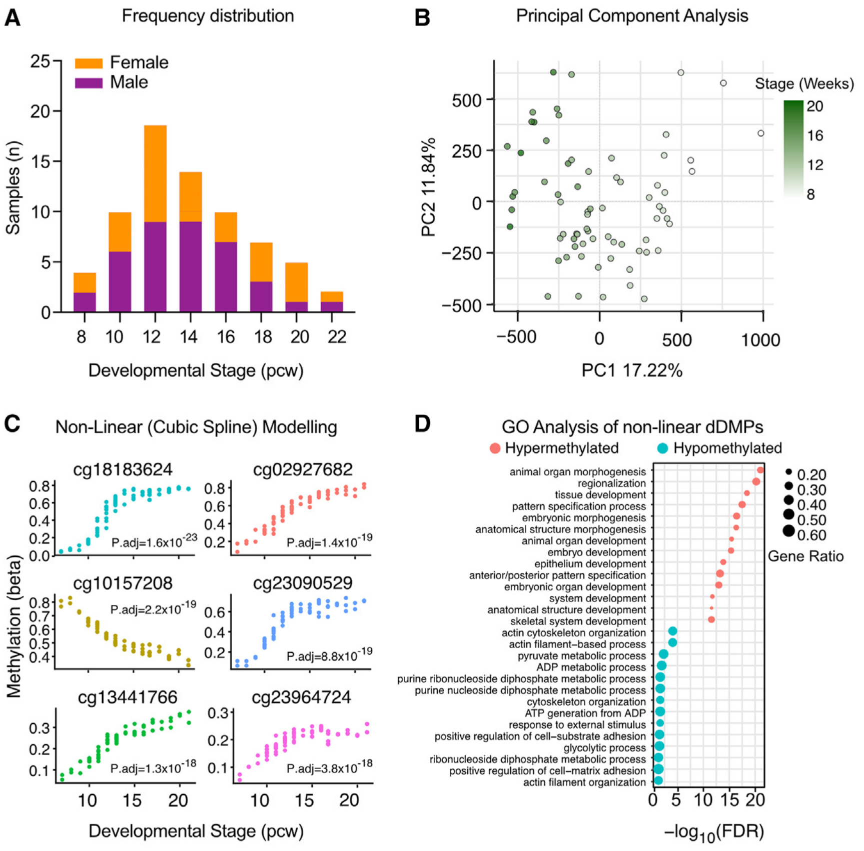

Figure 1. DNA methylation significantly changes at individual CpGs in fetal knee cartilage across the 7 to 21 pcw developmental window

(A) Histogram of samples ($n = 71$) showing the distribution of developmental stage (pcw) and sex. Purple, male; orange, female.

(B) Principal-component (PC) analysis of the samples revealed that methylation PC1 is associated with the developmental stage.

(C) The top six developmental differentially methylated probes (dDMPs) by non-linear (cubic spline) regression. P.adj, Bonferroni-adjusted p value.

(D) The top ten (by FDR significance) gene ontology (GO) biological process terms enriched among the non-linear hypermethylated (red) and hypomethylated dDMPs (blue).

examined with both Pearson correlation and a full regression model using the R functions cor and lm, respectively. Linear regression models included sex, batch, maternal age, maternal BMI, tissue weight, DNA concentration, and A_{260}/A_{280} ratio as covariates, similar to the DMP analysis. Resultant p values were adjusted by Benjamini-Hochberg correction.

To explore non-linear relationships between developmental stage and methylation, natural cubic splines with 5 degrees of freedom were incorporated in the limma model. The mean of the spline coefficients was used as a contrast to test for a developmental stage effect.

Differentially methylated regions (DMRs) were identified with dmrff R package (v.1.1.0) using the limma differential methylation results (modeling developmental stage as linear) as input. DMRs with >1 CpG and a Bonferroni-adjusted p value of <0.05 were considered significant. DMR overlap analysis was performed using the intersect function of the bedtools package (v.2.31.1) on assay for transposase-accessible chromatin (ATAC) regions derived from fetal knee samples or a reduced set of five Roadmap chondrocyte chromatin states.[21,29]

Further details of QC and analysis of DNAm datasets is included in supplemental methods.

Transcript expression analysis measured by real-time qPCR

RNA (1 μg) was reverse transcribed with the SuperScript IV cDNA synthesis kit (Invitrogen). The cDNA product was then used for real-time qPCR analysis following 1:20 dilution. Gene expression was quantified using TaqMan chemistry (QuantStudio3, Thermo Fisher). Pre-designed TaqMan assays (Integrated DNA Technologies, Belgium) were used to measure the expression of the target genes (Table S2). Gene expression was analyzed relative to the expression of three housekeeping genes, 18S, GAPDH, and HPRT1, using the $2^{-\Delta Ct}$ method as previously described.[11] The relationship of the methylation M values for CpGs within significant developmental DMRs (dDMRs) to the expression levels of annotated genes was

mQTL analysis

mQTL analysis was performed using the matrixEQTL R package (v.2.3). Using Sentrix ID (batch) and sex as covariates, we tested for *cis* associations based on a distance threshold of 500 kb.[30] To account for ancestry, five genotyping principal components (genotyping PCs) from the 1,000 Genomes PC analysis (genotyping PCA) were included in the mQTL analysis, as selected by the elbow method using PCAForQTL. To account for potential unknown technical confounders in the methylation data, ten methylation PCs were selected from the elbow method for inclusion in the mQTL modeling (Figure S4). Developmental stage was well explained by PC1 (Pearson correlation coefficient 0.92) and so was not explicitly included in the mQTL model to avoid redundancy. The mQTL model used was therefore methylation ~ genotype + 5 genotyping PCs + 10 methylation PCs + sex + batch. Resultant mQTLs were filtered based on a false discovery rate (FDR) < 0.05 or a conservative Bonferroni correction calculated using the matrixEQTL-reported total number of statistical tests. Significant mQTLs were associated with the nearest protein-coding gene, based on EnsDb (v.75) annotations.[31]

Colocalization analysis

We tested colocalization of 100 independent genome-wide significant SNVs previously associated with 11 OA skeletal-site phenotypes with the mQTL data.[32] Summary statistics for each phenotype were obtained from the musculoskeletal knowledge portal (https://msk.hugeamp.org/). To assess mQTLs within a range of ±500 kb from independent OA risk loci signal SNVs, we repeated the mQTL analysis while retaining all mQTL results (pvOutputThreshold = 1). For methylation probes within ±500 kb of the risk loci, we tested

associated SNVs for colocalization with genetic variants found in both the *cis* mQTL and the GWAS data. Colocalization analyses were performed separately for each GWAS phenotype using the coloc R package[33] (v.5.1.0) and the coloc.abf function, which calculates posterior probabilities (PPs) for five hypotheses:

H0: neither trait has a genetic association in the region
H1: only OA has a genetic association in the region
H2: only methylation has a genetic association in the region
H3: both OA and methylation are associated but with different causal variants
H4: both traits are associated and share a single causal variant

A PP of H4 >0.8 was considered as evidence for colocalization. We further filtered results to retain SNVs present in our imputed genotype data and where the mQTLs were significant (FDR < 0.05).

Results

The DNA methylome remains dynamic throughout cartilage development

Our first objective was to analyze the changing cartilage methylome throughout human skeletal development. Within the 71 cartilage samples retained post QC (ranging from 7 to 21 pcw), which contain only one cell type (the chondrocyte), we extracted both DNA and RNA. Representative images of developing limb skeletal preparations at 8–16 pcw are presented in Figure S5A. We obtained sufficient RNA from 47 of the isolated cartilage samples (ranging from 10 to 21 pcw) to enable gene-expression analysis of chondrocyte markers. The expression of chondrocyte progenitor markers *GDF5* and *SOX9* showed a significant decrease in expression correlating with increasing developmental stage ($p = 0.011$ and 0.006, respectively; Figures S5B and S5C). Additionally, the expression of articular chondrocyte marker *PRG4* significantly increased with stage ($p = 0.003$; Figure S5D). The type II collagen gene, *COL2A1*, was highly expressed throughout the stages and did not significantly change ($p = 0.219$; Figure S5E). This confirmed that we had accurately dissected chondrocyte populations within our bulk tissue analysis.

Analysis of DNA methylation (DNAm) within all 71 samples identified 19,070 significant developmental differentially methylated probes (dDMPs; Bonferroni-adjusted $p < 0.05$, $\log_2$ fold change >0.1; Table S3), with 66% becoming significantly hypomethylated through skeletogenesis. We analyzed the profiles of differential methylation across the developmental stages, identifying three clusters. CpGs within clusters 1 and 2 became significantly hypomethylated across the developmental window (Figure S6, red and blue, respectively). Conversely, the CpGs within cluster 3 (Figure S6, green) became hypermethylated throughout development. Gene ontology (GO) analysis revealed significant biological process (BP) terms (FDR < 0.05) for cluster 3 including "embryonic morphogenesis" (FDR = 3.97×10^{-9}) and "mesenchyme development" (FDR = 1.21×10^{-8}; Table S4). This indicated that dDMPs becoming hypermethylated mapped to early transcriptional regulators of skeletal development. This soft clustering approach did not reveal any complex patterns of DNAm change in the investigated samples. Therefore, to further explore non-linear relationships, we applied an additional differential methylation analysis, whereby we modeled developmental stage using natural cubic splines. This similarly identified 22,073 significant dDMPs (Bonferroni-adjusted $p < 0.05$; Table S5). Similarly, no complex patterns of differential DNAm were identified, even among the most significant CpGs (Figure 1C). Comparable to cluster 3 (above), GO analysis of the hypermethylated dDMPs showed enrichment for 451 BP terms including "regionalization" (FDR = 9.68×10^{-21}) and "skeletal system development" (FDR = 2.68×10^{-12}; Figure 1D and Table S6). Within the hypomethylated CpGs, enrichment of 14 BP terms (with FDR < 0.05) was identified including "actin filament-based process" (FDR = 8.37×10^{-5}) and "positive regulation of cell-matrix adhesion" (FDR = 0.04; Figure 1D and Table S6).

To gain further insight into the biological role of the DMPs, we next investigated the presence of DMRs based upon regions of DNAm coregulation. We identified 7,862 significant (Bonferroni-adjusted $p < 0.05$) dDMRs ranging in size between 2 and 53 CpGs (mean = 2.8) and consisting of 22,495 individual CpGs (Table S7). In Figure 2A, the most significant dDMRs (irrespective of size) are annotated with the nearest protein-coding gene. The largest hypermethylated dDMRs mapped to genes encoding known transcriptional regulators of early development including the *HOXA* gene locus and *TBX5* (Figure 2B). GO analysis revealed 521 significant (FDR < 0.05) BP terms associated with hypermethylated dDMRs (Table S8). The top 30 most significant biological process terms included "skeletal system development" (FDR = 1.46×10^{-16}), "anatomical structure development" (FDR = 8.40×10^{-19}), and "embryonic morphogenesis" (FDR = 9.13×10^{-22}; Table S8 and Figure 2C). Just 21 biological processes were significantly associated with hypomethylated regions (FDR < 0.05; Table S9), which mapped to genes encoding extracellular matrix (ECM) proteins including *TNXB* (Tenascin-X) and *SPON2* (Spondin-2; Figure 2B). "Response to external biotic stimulus" was the most significantly enriched BP term (FDR = 1.79×10^{-5}). Several terms were further identified relating to matrix synthesis, including BP "supramolecular fiber organization" (Figure 2C), molecular function "extracellular matrix constituent" (FDR = 5.11×10^{-5}), and cellular compartment "extracellular matrix" (FDR = 2.95×10^{-8}) (Table S9).

Developmental DMRs overlap with open chromatin regions and correlate with gene expression

We next intersected the physical location of the dDMRs with open chromatin peaks from 12 pcw fetal distal femoral cartilage. We identified overlap between 2,949 dDMRs (35.6%), mapping to 2,153 genes, further highlighting those most likely to impart regulatory function

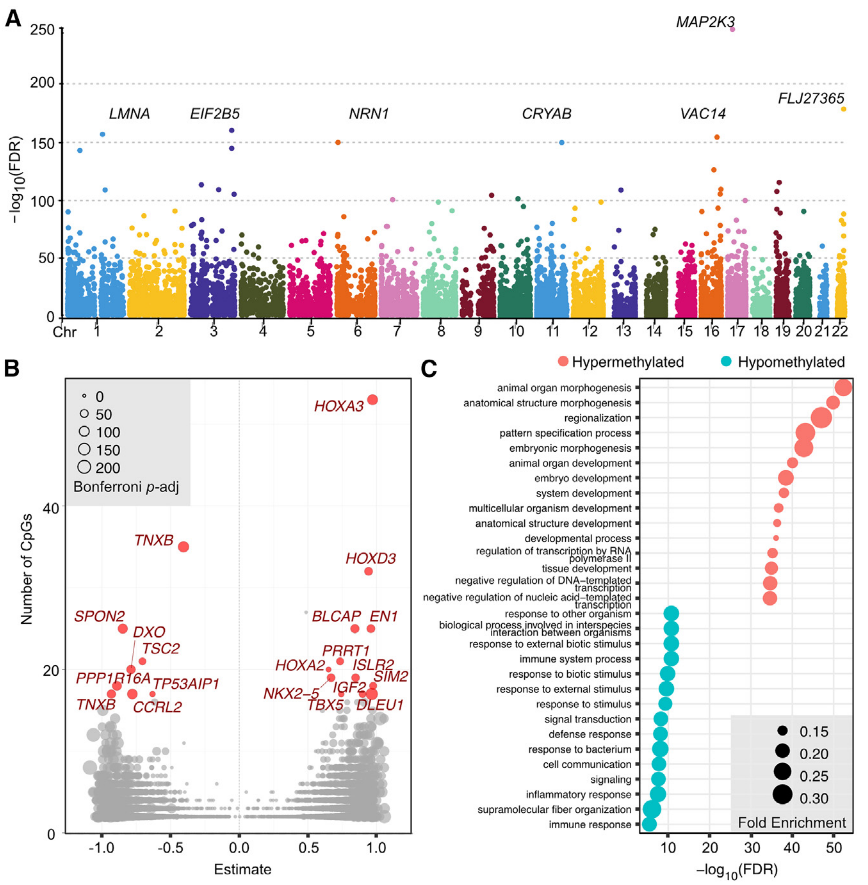

Figure 2. Co-regulation of DNAm at individual CpGs results in developmental differentially methylated regions (dDMRs) in fetal knee cartilage

(A) Manhattan plot of dDMRs across the epigenome. The nearest protein-coding gene to the most significant dDMRs is labeled.

(B) Volcano plot of dDMRs by size (number of CpGs). The largest dDMRs are highlighted in red with the nearest protein-coding gene labeled.

(C) GO analysis of the most significant biological processes enriched in hypermethylated (red) and hypomethylated (blue) dDMRs.

during this window of development (Table S10). The largest hyper- and hypomethylated regions overlapping with fetal knee ATAC peaks are displayed in Table 1.

To investigate the putative functional role of these DMRs in developmental gene regulation, we measured the expression of eight of the genes mapping to the largest dDMRs. Six measured transcripts mapping to hypermethylated regions (*MEIS1*, *EN1*, *HOXD3*, *IRX1*, *HOXA3*, and *SIM2*) were expressed at low levels throughout the captured developmental window (Figure S7, red). No significant relationships (adjusted p value [p.adj] $>$ 0.05) were identified between gene expression and DNAm within the hypermethylated dDMRs through either direct correlation or linear regression modeling (Table S11).

We measured expression of three genes mapping to the largest hypomethylated regions, *CCRL2* (dDMR 1894), *SPON2* (dDMR 2329), and *TNXB* (dDMRs 3370 and 3378). Of these three genes, the two with known biological roles in cartilage biology (*SPON2* and *TNXB*) were expressed at much higher levels in the fetal chondrocytes compared to the other seven measured genes (Figure S7, blue). The increased expression of these two genes significantly correlated with decreasing methylation within the mapped dDMRs at 21/25 ($p = 3.9 \times 10^{-4} - 0.044$), and 15/52 ($p = 9.2 \times 10^{-5} - 0.028$) CpGs, respectively (Figure 3). Following multiple test correction, no significant associations were found using the linear regression model.

Developmental DMRs are enriched in active enhancers and transcription factor binding sites

We performed motif enrichment analysis on the hyper- and hypomethylated dDMRs for enrichment of known TF binding motifs. Using both the hyper- and hypomethylated dDMRs, binned by $\log_2$ fold change (Figure S8), we identified significant enrichment (FDR $\leq$ 0.05) of 46 known TF motifs on the HOCOMOCO database[34] (Figure 4A and Table S12). Notably, the regions that decreased in methylation across the developmental window were enriched for TFs with known roles in chondrocyte biology and synovial joint formation including NFATC1 (p.adj $=$ 0.03)[35] and the FOS/JUN families of TFs (p.adj$< 2.6 \times 10^{-14}$). The regions that increased in methylation across development were enriched for motifs of early transcriptional regulators of skeletogenesis, including HOXD10 (p.adj $= 0.00024$)[36] and RUNX2/3 (p.adj < 0.05; Figure 4A).[37]

Finally, we overlapped the dDMRs with Roadmap chromatin state data generated in human chondrocytes to test for enrichment among distinct categories of CREs. Hypermethylated dDMRs were significantly over-represented (FDR $<$ 0.05) in promoters (transcription start site [TSS], red; fold enrichment 1.36), transcribed (green; fold enrichment 1.22), and repressed regions (gray; fold enrichment 1.07; Figure 4B) when compared to all identified significant dDMRs. Conversely, hypomethylated dDMRs were significantly over-represented (FDR $<$ 0.05) in enhancers (yellow; fold enrichment 1.11) and under-represented in transcribed (green; fold enrichment 0.85) and promoter regions (TSS, red; fold enrichment 0.75; Figure 4B), indicating that across development, regions becoming hypomethylated were predominantly regulating the function of gene enhancers.

Chondrocyte DNA methylation in articular cartilage development exhibits sexual dimorphism

Previously, methylome analysis performed in 358 blood samples from extremely premature neonates (at 23–27

Table 1. **Top developmental DMRs that intersect with open chromatin regions in developing knee cartilage**

DMR ID	Chr	Start	End	Gene	Classification/function	Bonferroni-adjusted *p* value	CpGs (*n*)
**Largest hypermethylated DMRs with 12 pcw ATAC overlap**							
1383	2	119605959	119611101	*EN1*	engrailed homeobox transcription factor	3.69×10^{-38}	25
1529	2	177022056	177029813	*HOXD3*	homeobox transcription factor	1.54×10^{-37}	32
2594	4	151500192	151503588	*LRBA*	lipopolysaccharide-responsive and beige-like anchor protein	1.61×10^{-45}	16
2715	5	3597311	3601762	*IRX1*	iroquois homeobox transcription factor	1.69×10^{-12}	16
3124	5	172660884	172664439	*NKX2-5*	homeobox transcription factor	1.85×10^{-36}	19
3390	6	32115979	32118204	*PRRT1*	transmembrane protein	6.56×10^{-26}	21
3879	7	27142100	27143788	*HOXA2*	homeobox transcription factor	7.23×10^{-6}	20
3894	7	27181418	27188020	*HOXA3*	homeobox transcription factor	4.76×10^{-76}	53
7309	15	74423308	74427499	*ISLR2*	LRR-domain-containing receptor	5.02×10^{-35}	19
9022	20	36145902	36148779	*BLCAP*	regulator of cell proliferation	7.76×10^{-44}	25
9220	21	38076709	38080975	*SIM2*	basic-helix-loop-helix transcription factor	2.51×10^{-26}	18
**Largest hypomethylated DMRs with 12pcw ATAC overlap**							
1894	3	46447254	46449636	*CCRL2*	chemokine receptor	4.76×10^{-69}	17
2329	4	1201588	1206150	*SPON2*	ECM glycoprotein	6.88×10^{-61}	25
3370	6	32013974	32016535	*TNXB*	ECM glycoprotein	1.88×10^{-84}	35
3378	6	32048679	32050927	*TNXB*	ECM glycoprotein	1.06×10^{-45}	17
4665	8	145727318	145729537	*PPP1R16A*	protein phosphatase subunit	1.60×10^{-56}	18
6043	11	128812804	128813688	*TP53AIP1*	apoptosis-inducing protein	1.59×10^{-9}	17
7501	16	2131718	2136269	*TSC2*	growth inhibitory protein	3.00×10^{-24}	21

The largest identified dDMRs (no. of CpGs >16) that became either hypermethylated (upper) or hypomethylated (lower) across the captured developmental window. The nearest physical gene to each dDMR is listed. Chr, chromosome; *p*.adj, Bonferroni-adjusted *p* value.

weeks' gestation, which relates to ~21–25 pcw) showed that a large proportion of CpGs exhibited sexually dimorphic levels of DNAm.[38] Using the EPIC array, Santos et al. identified 5,595 DMPs (FDR < 0.00001), 95% of which were hypermethylated in females.[38] We next tested for significant sex-specific differences in DNAm among our developmental cartilage samples. Epigenome-wide analysis revealed 143 sex-specific DMPs (sDMPs; Bonferroni-adjusted $p < 0.05$), mapping to 119 genes (Table S13 and Figure S9A). Of these sites, 90 (62.9%) were hypermethylated in the female samples. Eighty-five percent (121) of the sDMPs identified in developing articular cartilage also showed significant sex effects in the neonatal blood dataset, and comparison of the effect sizes showed a strong correlation ($R = 0.91$, $p < 2.2 \times 10^{-16}$; Figure S9B). There was a direct overlap between all the top 20 reported probes, with methylation effects occurring in the same direction (Table 2 and Figure S9C). Among the genes mapping to these CpGs were *NAB1*, *RFTN1*, and *CEP170*.

We further identified 102 significant sDMRs (Bonferroni-adjusted $p < 0.05$), consisting of 178 CpGs (Table S14). At 84 identified regions (82%), DNAm was significantly higher in the female samples than in male samples. No significant GO terms (all FDR > 0.05) were associated with either the sDMPs or sDMRs, consistent with the findings of Santos et al. in their analysis of neonatal blood samples.[38]

mQTLs colocalizing with OA risk signals are active during articular cartilage development

Emerging evidence increasingly supports developmental origins of OA risk in later life.[21,39,40] Our second main objective in this study was to investigate the presence of mQTLs in the developing joint cartilage and test for colocalization with OA GWAS signals. To enable colocalization analysis, we first carried out epigenome-wide analysis in our samples, which revealed 474,356 significant (Bonferroni-adjusted $p < 0.05$) *cis*-mQTLs (individual SNV-CpG associations; Table S15). The signals were widespread across the epigenome (Figure 5A) and consisted of 14,801 individual CpGs and 309,602 SNVs. The most significant mQTLs mapped to the genes *CPEB4* (p.adj $= 3.9 \times 10^{-42}$), *PHGDH* (p.adj $= 4.0 \times 10^{-47}$), and *ELM3* (p.adj $= 1.0 \times 10^{-39}$).

We next compared our epigenome-wide mQTLs to other reported human fetal (brain)[41] and cartilage (adult low-grade knee OA)[8] datasets (Figure S10). Both studies

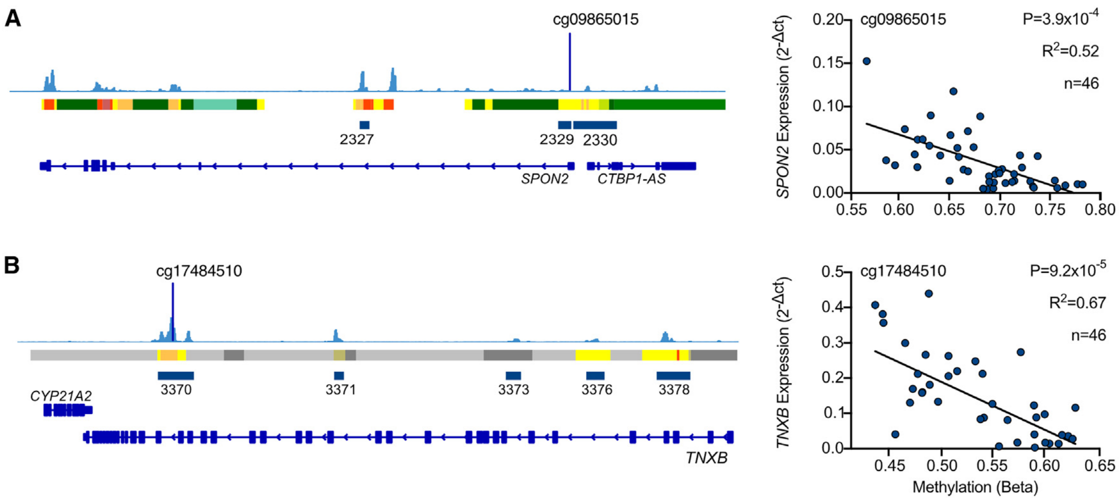

Figure 3. The expression of genes mapping to dDMRs significantly correlates with methylation within the regions

(A) Screenshot of the Integrated Genome Viewer (IGV) displaying the *SPON2* gene locus on chromosome 4. Fetal knee cartilage ATAC-seq peaks (light blue) show regions of open chromatin. Below the peaks, the ROADMAP chromatin state in human cultured chondrocytes (E049) is displayed. Red, active transcription start site; yellow/orange, enhancer; green, transcribed; gray, repressed. Hypomethylated dDMRs are shown in dark blue and annotated with the dDMR ID. Genes within the region are shown in dark blue with the gene names labeled. Gene expression of *SPON2* measured by real-time qPCR is plotted against DNAm (beta) at cg09865015, which falls within dDMR 2329.

(B) As in (A) for the *TNXB* locus on chromosome 6. Cg17484510 falls within dDMR 3370.

Statistical analysis was performed by Pearson correlation using methylation M values. Beta values are displayed for ease of interpretation. *p* values are adjusted with Benjamini-Hochberg correction.

had employed the Illumina 450K array to generate the reported data, so we filtered our significant mQTLs (p.adj < 0.05) by those also present on the historical array before testing for overlap between the datasets. In the fetal brain dataset (173 samples ranging from 8 to 24 pcw), 29.7% of the fetal cartilage mQTLs were present, compared to 52% in the adult (OA) cartilage dataset (Figures S10A and S10B). Comparison of the effect sizes between the cross-tissue mQTLs revealed strong correlations ($R = 0.92$ and 0.94 in the fetal brain and adult cartilage, respectively; Figures S10C and S10D).

To identify shared impacts of SNV genotype upon OA risk and DNAm, we next performed a colocalization analysis of 100 risk signals from 11 OA phenotypes with the fetal cartilage mQTLs. This analysis utilized the summary statistics from the largest OA GWAS published to date, which included genotype data from 826,690 individuals spanning nine distinct populations.[32] In total, we identified 102 colocalizations with significant (p.adj < 0.05) fetal cartilage mQTLs (PP > 0.8; Table S16) among 46 CpGs spanning 24 genomic loci (Table 3). mQTLs colocalizing with OA risk signals (OA-mQTLs) were identified at eight loci that have previously been reported in investigations of aged adult cartilage. We overlapped the physical location of the CpGs with open chromatin regions in fetal and OA knee chondrocytes[21] and regions of H3K27ac enrichment in fetal limb tissues.[42] Evidence supporting function was identified at 15 loci, with around half (eight) consisting of mQTLs that fell within open chromatin or H3K27ac-enriched regions solely in developmental samples. Using chondrocyte chromatin state data, we further identified that the mQTLs were significantly depleted ($p = 0.008$) in chondrocyte-repressed regions (0.58-fold) Figure 5A) and enriched in enhancer (1.51-fold) and transcribed (1.53-fold) regions, although not to a statistically significant degree ($p = 0.18$ and 0.07, respectively).

One example of a previously reported OA-mQTL is on chromosome 15, where the mQTL at cg12031962 maps to *ALDH1A2* ($p = 2.2 \times 10^{-10}$). This mQTL colocalizes with the primary phenotypes “Hand OA” (PP = 0.99, Figure 5C), “Thumb OA” (PP = 0.99), “Total Knee Replacement” (PP = 0.98), and “Finger OA” (PP = 0.93; Table S16) marked by the risk SNV rs11071366 (A>T). Here, the major and OA effect allele, A, correlates with an increase in DNAm at the intronic CpG (Table 3).[8]

Conversely, on chromosome 20, a single intronic mQTL was identified, which maps to a previously unreported CpG at a known OA risk locus, mapping to the gene *GDF5* (Figure 5D). Here, an mQTL at cg12548730 colocalized with the OA risk SNV rs143384 in the “All OA” (PP = 0.913), “Total Joint Replacement” (PP = 0.912), and “Knee and Hip OA” (PP = 0.895) primary phenotypes (Table S16). The CpG is located within an intron of *UQCC1*, downstream of previously reported *GDF5* cartilage enhancers R3 and R4.[40,45] The OA effect allele (G) significantly correlated with lower levels of DNAm ($p = 7.6 \times 10^{-6}$; Figure 5D). Analysis of our existing knee cartilage ATAC-sequencing (ATAC-seq) dataset in human distal femur (knee) and adult knee cartilage samples showed that the CpG is located adjacent to an open chromatin peak in fetal cartilage (blue) but not

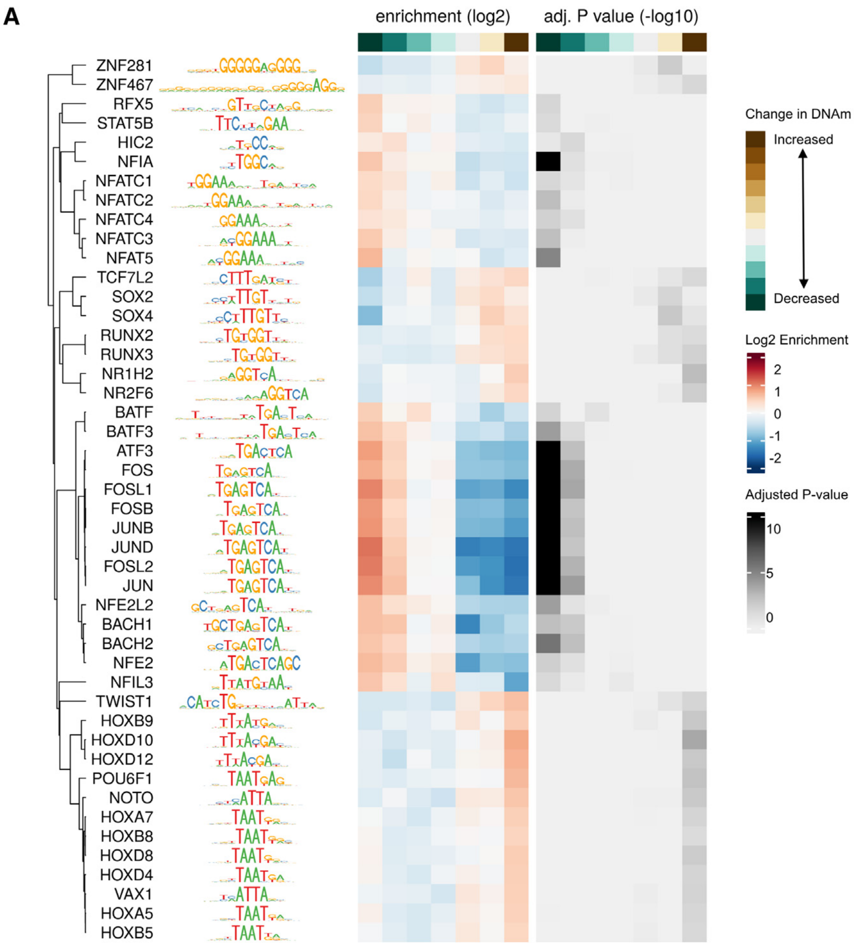

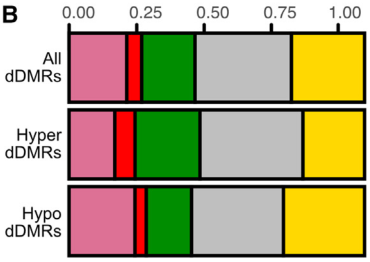

Test	Feature	FDR	Enrichment
Hypermethylated dDMRs	Flanking TSS	2.57×10^{-7}	0.79
	TSS	8.44×10^{-5}	1.36
	Transcribed	9.61×10^{-7}	1.22
	Repressed	0.01	1.07
	Enhancer	4.19×10^{-6}	0.84
Hypomethylated dDMRs	FlankingTSS	8.10×10^{-5}	1.14
	TSS	0.0004	0.75
	Transcribed	3.85×10^{-5}	0.85
	Repressed	0.054	0.95
	Enhancer	0.0004	1.11

Figure 4. Developmental DMRs are enriched in regulatory elements

(A) Transcription factor (TF) motifs taken from the HOCOMOCO database, which are enriched in dDMRs. Left: name and logo plot of enriched TF motif within the dDMRs, Center: heatmap of $\log_2$ enrichment value of the motifs within hypermethylated (brown) or hypomethylated (green) dDMRs. Right: heatmap of $-\log_{10}$ adjusted p value. Darker color indicates higher statistical significance.

(B) Frequency of identified dDMRs overlapping with human cultured chondrocyte (Roadmap E049) chromatin states. Left: barplot of proportions of dDMR overlap with one of five chromatin states. Hyper, hypermethylated; hypo, hypomethylated. The colors used for labeling on each bar plot are shown in the following order (left to right): region flanking transcription start site (TSS), pink; TSS, red; transcribed region, green; repressed region, gray; enhancer, yellow. Right: statistical significance was calculated using Fisher's exact test with Bonferroni correction, with all significant DMRs as background. Significant under- or over-representation is labeled with the adjusted p value.

in OA cartilage (orange). This region has not previously been investigated as a regulatory element for *GDF5*. A previous mQTL at this locus has been reported in adult OA cartilage, located adjacent to the promoter of *UQCC1* (cg14752227).[11]

At the majority of the loci (16), no previous OA-mQTLs have been reported in analyses of adult cartilage. On chromosome 10, three OA-mQTLs were identified that fell within putative regulatory elements of *CHST3*, encoding carbohydrate sulfotransferase 3 (Figure 5E). Two of the CpGs, cg15093997 and cg20048045, are located within the 3′ UTR of the gene, near to open chromatin regions. At both CpGs, the effect allele, A, at rs3740129 (G>A) correlated with significantly decreased levels of DNAm ($p = 6.8 \times 10^{-13}$ and 7.4×10^{-8}, respectively). Conversely, at the more distal CpG (cg17209404), located within an intergenic open chromatin region only in fetal cartilage, DNAm was significantly increased in the presence of the OA effect allele ($p = 1.4 \times 10^{-5}$; Table S16).

Genetic and epigenetic effects influence distinct subsets of CpGs comprising the fetal cartilage methylome

Finally, we tested whether the 14,801 CpGs that comprise the identified significant mQTLs across the epigenome are amenable to external influences occurring between sexes (143 sDMPs) and through articular cartilage development (19,070 dDMPs). Between the mQTLs and dDMPs, an overlap of just 182 CpGs was identified, indicating that only 1% of CpGs that are significantly regulated by DNA sequence are impacted by developmental stage ($p = 8.31 \times 10^{-40}$). Similarly, there was no overlap observed between the mQTL CpGs and the 143 sDMPs ($p = 0.0426$). This suggests that the CpGs at which DNAm is heavily influenced by the genetic architecture of the region are less amenable to environmental influences on the epigenome.

Discussion

In this study, we present the epigenomic landscape across the development of human articular cartilage. This analysis provides valuable insight into chondrocyte methylomic plasticity across a large window of human

Table 2. Top 20 sDMPs in developing articular cartilage

CpG ID	Chr	Position	Log_2 fold change	Bonferroni-adjusted p value	Nearest gene	Mean DNAm male	Mean DNAm female
cg03618918	1	160865097	0.69918938	3.62×10^{-19}	*ITLN1*	0.75378011	0.651373
cg26919182	1	202522232	−1.3731547	3.14×10^{-24}	*PPP1R12B*	0.32815365	0.55040867
cg15817705	1	209406063	0.88609364	1.16×10^{-19}	*CAMK1G*	0.74307904	0.60401846
cg12691488	1	243053673	3.36115528	2.50×10^{-42}	*CEP170*	0.28244637	0.03553781
cg15228509	1	243074717	−0.8995054	1.20×10^{-37}	*CEP170*	0.39543316	0.54319268
cg02989351	2	9770584	−0.7794246	1.17×10^{-21}	*YWHAQ*	0.09035353	0.14518659
cg03226871	2	128128958	0.47034637	6.72×10^{-18}	*MAP3K2*	0.58320181	0.49970286
cg19765154	2	191524409	1.01633412	1.39×10^{-33}	*NAB1*	0.78662084	0.64093121
cg20262915	2	191524489	0.51673912	2.45×10^{-23}	*NAB1*	0.51381524	0.42198249
cg00148935	3	16398839	−0.936831	2.16×10^{-26}	*RFTN1*	0.43815513	0.59589401
cg02758552	3	49395714	−0.5989841	7.38×10^{-18}	*GPX1*	0.28510191	0.37961935
cg26516287	7	12629275	−0.5857036	5.71×10^{-15}	*SCIN*	0.6857675	0.76416104
cg07850329	9	33265029	−0.6365864	4.53×10^{-21}	*CHMP5*	0.21186414	0.29431552
cg07852945	9	84303915	−1.401265	4.09×10^{-17}	*TLE1*	0.04339289	0.11272387
cg02716779	9	131016143	−0.5731337	1.35×10^{-21}	*DNM1*	0.53573475	0.62982978
cg26355737	13	114292172	0.86948882	3.32×10^{-19}	*TFDP1*	0.8139748	0.70371779
cg11284736	15	83826708	0.68755673	2.07×10^{-19}	*HDGFRP3*	0.75872287	0.65989451
cg03626220	16	71052200	0.58799107	1.29×10^{-22}	*HYDIN*	0.64907414	0.55169401
cg03218192	17	33914403	−0.6129854	1.98×10^{-18}	*AP2B1*	0.29516199	0.3882843
cg12607525	17	42286849	0.46716174	2.39×10^{-20}	*UBTF*	0.60081041	0.52037461

The most significant DMPs between male and female cartilage samples are listed. Chr, chromosome. Effect size is indicated by $\log_2$ of the fold change in DNA methylation (DNAm) when comparing male ($n = 36$) to female ($n = 35$) samples.

skeletogenesis. We identified that developmental stage is the predominant determinant of methylation status within our samples, with ~3% of CpGs significantly changing across the captured time frame (Bonferroni-adjusted $p < 0.05$). We further identified >8,200 dDMRs, 36% of which intersected with open chromatin regions in fetal knee cartilage (12 pcw), providing evidence of function. We demonstrated that 143 of the captured CpGs exhibited sexually dimorphic effects, with the majority (63%) being hypermethylated in female samples. Finally, we conducted an epigenome-wide mQTL analysis and found strong evidence of colocalization of these molecular traits with genetic drivers of multiple OA phenotypes, building upon existing evidence of the developmental origins of a complex genetic disease of older age.

In 2023, Zhang et al. published the first single-cell and spatial transcriptomic atlas of the developing human hindlimb,[46] providing evidence of nascent articular chondrocytes that expressed high levels of *PRG4* in the distal femur by 8 pcw. In our data, the considerable impact of the developmental stage upon the cartilage methylome partly reflects the changing nature of the chondrocyte phenotype captured in our bulk analysis. At earlier stages (<12 pcw) we have captured epiphyseal chondrocytes along with the nascent articular cartilage that will continue to develop postnatally, whereas after 12 pcw, we were able to isolate the nascent articular cartilage more specifically. Our gene-expression profiling data demonstrate that the primary population of cells analyzed in this study is nascent epiphyseal and articular chondrocytes. Underscoring this, our data also align with studies of embryonic development of epiphyseal and articular cartilage in mouse models, where time-course expression and lineage-tracing studies have shown that both *Gdf5* and *Sox9* expression decreases, *Col2a1* remains relatively stable, and *Prg4* increases as joint development proceeds beyond cavitation.[47–49] In 2015, Spiers et al. applied the Illumina HumanMethylation450 array to DNA extracted from 179 human fetal brain samples between 8 and 26 pcw.[50] They identified that DNAm at ~7% of captured CpGs significantly changed across the developmental window, the higher degree of significant change potentially reflective of a more heterogeneous cell population within the developing brain tissue.

The largest hypermethylated dDMRs identified in this study predominantly map to transcriptional regulators of early embryogenesis and skeletal patterning, including *MEIS1*, *EN1*, *IRX1*, and *HOXA3*. No significant relationships were identified between the expression of any of

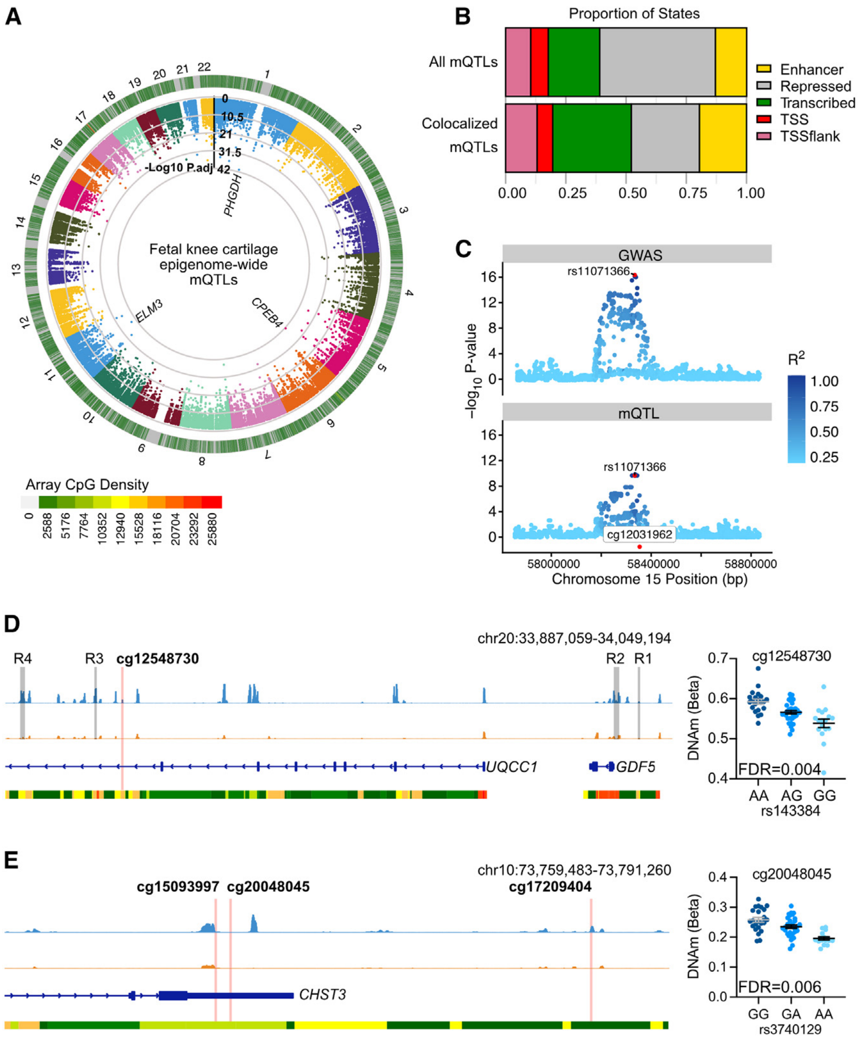

Figure 5. Epigenome-wide methylation quantitative trait locus (mQTL) analysis reveals colocalization with OA genetic risk signals in developmental cartilage

(A) Manhattan plot of significant (Bonferroni-adjusted p value <0.05) epigenome-wide mQTL signals. The inner heatmap represents the Illumina probe density in the respective regions across the genome. The genes mapping to the top three (most significant) mQTLs are displayed.

(B) Barplot of enrichment analysis of epigenome-wide versus trait-specific (OA) mQTLs in chondrocyte chromatin states. The colors used for labeling on each barplot are shown in the following order (left to right): region flanking TSS, pink; TSS, red; transcribed region, green; repressed region, gray; enhancer, yellow.

(C) An example of a colocalization event. The mQTL for the methylation site cg12031962 (bottom) colocalized with the GWAS signal rs11071366 for hand OA in the same genomic region (top). Here, we observed a posterior probability for a shared causal variant of 99%.

(D and E) IGV screenshot of the genetic region mapping to fetal cartilage mQTLs colocalizing with OA GWAS signals rs143384 (D) and rs3740129 (E). Light blue, fetal distal femoral cartilage (knee) ATAC-seq data with peaks representing open chromatin regions; orange, adult low-grade OA knee cartilage ATAC-seq data; dark blue, gene transcript; bottom track is Roadmap chromatin state data from E049 cultured chondrocytes. Red, active TSS; yellow/orange, enhancer; green, transcribed; gray, repressed.

these genes and DNAm in the identified regions. However, the expression of these genes was low in all samples from 7 pcw onward, and we postulate that we have captured a developmental window beyond the time frame in which these genes impart their functional roles in cartilage development. Here, we appear to be observing hypermethylation of DNA as these gene regions become repressed, potentially to reinforce chromatin condensation. This is supported by the results of the GO analysis, which are enriched for a plethora of biological-process terms relating to embryogenesis and skeletal development. Furthermore, the hypermethylated dDMRs were over-represented in repressed regions of mature chondrocytes. These observations also align with functional studies in murine models of limb development and the recent spatial maps of gene expression in human limb development.[46] *MEIS1* is a regulator of proximodistal identity[51,52] and was further shown by Zhang et al. to be expressed specifically in the proximal hindlimb (developing femur) at 5.6 pcw.[46] *En1* and *Irx1*, which orchestrate early murine limb bud patterning and digit formation, respectively, are most highly expressed earlier and in the most distal locations in the limb.[53]

Conversely, several large hypomethylated regions mapped to genes encoding ECM proteins including Spondin-2 and Tenascin-X, highlighting the capability of the DNA methylome to underlie transcriptional changes. We investigated the expression of three genes mapping to the largest significant hypomethylated dDMRs, identifying that the two encoding known ECM components (*SPON2* and *TNXB*) significantly increased in correlation with decreasing DNAm. A more complex regression model failed to identify significant relationships between DNAm in the DMRs and expression of the matched transcripts, although this is perhaps unsurprising because while our dataset was adequately powered to detect changes in DNAm, it is a relatively modest sample size for detection of gene-expression changes in human populations, which often require much larger cohorts to identify such correlative effects.[54] Across the developmental window, hypomethylated dDMRs were enriched in annotated chondrocyte enhancers. This is consistent with earlier reports using a mesenchymal stem cell *in vitro* model, which demonstrated that throughout chondrogenic differentiation, hypomethylation of DNA is enriched in enhancer regions.[55]

Table 3. OA genetic risk loci colocalizing with significant mQTLs in human fetal knee cartilage

Colocalization analysis									Studies of OA cartilage		Functional annotation		
**Chr**	**SNV**	**CpG**	**Gene (CpG)**	**Coloc. PP (H4)**	**SNV alleles**	**OA effect allele (EA)**	**MAF (EUR)**	**EA impact on DNAm**	**Locus identified? (gene)**	**Same CpG?**	**H3K27ac fetal limb**	**ATAC peak fetal**	**ATAC peak OA**
2	rs74676797	cg22925888	*TMEM18*	0.81	A>G	A	0.17	+	N	N/A	Y	N	N
		cg06162668		0.81				+			Y	N	N
3	rs3774354	cg00150837	*GNL3*	0.80	G>A	A	0.40	–	Y[11,43] (*GNL3*)	N	N	N	N
		cg25917518	*ITIH1*	0.98				+			N	N	Y
		cg06706579		0.96				+			N	N	N
		cg18404041		0.95				–		Y[43]	N	N	N
4	rs1530586	cg18815463	*TACC3*	0.93	T>C	T	0.22	+	Y[8,13,43]	N	N	N	N
		cg05874882		0.95				–		Y[8]	N	N	N
4	rs11729628	cg07870920	*PRDM5*	0.86	G>T	T	0.20	–	N	N/A	N	N	N
5	rs10062749	cg01030629	*SPRY4*	0.99	G>T	T	0.26	–	N	N/A	N	N	Y
		cg15672022		0.94				–			Y	Y	Y
		cg16740022		0.90				–			Y	Y	Y
5	rs3884606	cg16278800	*FGF18*	0.98	A>G	A	0.47	–	N	N/A	N	Y	Y
6	rs9396861	cg04025088	*RNF144B*	0.99	A>C	A	0.41	–	N	N/A	Y	Y	N
6	rs79220007	cg00854024	*HFE*	1.00	T>C	T	0.04	–	N	N/A	Y	N	N
6	rs2856821	cg17362900	*HLA-DPB1*	0.97	T>C	T	0.23	–	Y[8,13,43]	N	N	Y	Y
		cg19053046		0.94				–		N	Y	Y	Y
		cg25045942		0.98				–		N	Y	Y	Y
6	rs2038740	cg25299750	*TCP11*	0.91	T>C	T	0.28	+	N	N/A	Y	N	N
		cg17246275		0.91				+			Y	N	N
9	rs62578126	cg16535956	*LMX1B*	0.88	C>T	T	0.37	+	N	N/A	N	N	N
10	rs3740129	cg15093997	*CHST3*	0.86	G>A	A	0.43	–	N	N/A	Y	N	Y
		cg20048045		0.98				–			Y	N	N
		cg17209404		0.96				+			Y	Y	N
11	rs3993110	cg25843174	*TEAD1*	0.95	A>C	A	0.42	–	N	N/A	Y	N	N
11	rs1517572	cg22678680	*METTL15*	0.92	C>A	A	0.41	+	N	N/A	N	N	N
		cg26656020		0.82				+			N	N	N

(*Continued on next page*)

Table 3. *Continued*

Colocalization analysis									Studies of OA cartilage		Functional annotation		
11	rs67924081	cg01366692	*SCYL1*	0.96	A>G	A	0.26	+	Y[43]	N	N	N	N
		cg17120908	*SSSCA1*	0.99				+		N	Y	Y	Y
12	rs7953280	cg19571390	*CRADD*	0.81	G>C	C	0.50	+	Y[8]	N	N	N	N
15	rs4380013	cg01701297	*USP8*	0.91	G>A	A	0.22	–	N	N/A	N	N	N
15	rs11071366	cg12031962	*ALDH1A2*	0.99	A>T	A	0.35	+	Y[11,43] (*ALDH1A2*)	Y[11,43]	Y	Y	Y
15	rs12908498	cg15164144	*SMAD3*	0.98	C>G	C	0.47	–	N	N/A	N	Y	N
		cg21106065		0.99				+			N	Y	N
19	rs10405617	cg16876852	*ILF3*	0.82	G>A	A	0.37	–	Y[8]	N	N	N	N
20	rs143384	cg12548730	*UQCC1*	0.91	T>C	T	0.41	+	Y[11,43] (*GDF5*)	N	N	Y	N
21	rs9981408	cg21864030	*ERG*	0.89	G>T	T	0.21	–	N	N/A	N	N	N
21	rs9981884	cg22249046	*PSMG1*	0.82	G>A	A	0.43	–	N	N/A	N	N	N
		cg11890956		0.81		A		+			Y	Y	Y
		cg05821552	*BRWD1*	0.98		A		–			N	N	N
		cg14227474		0.89		A		–			N	N	N
22	rs12160491	cg17109681	*TRIOBP*	0.98	A>G	A	0.33	–	N	N/A	Y	Y	N
		cg06521852		0.96		A		–			Y	N	N
		cg11795195		0.97		A		–			Y	N	N
		cg25404088		0.91		A		–			Y	Y	Y
		cg26028571		0.92		A		–			N	N	Y

For colocalization analysis, the nearest gene to the CpG is listed. H4 PP, posterior probability that both traits are associated and share a single causal variant; MAF (EUR), minor allele frequency in European populations. EA Impact on DNAm indicates whether the effect allele is associated with an increase (+) or decrease (−) in DNAm. For mQTL analysis of OA cartilage, we investigated whether any of the colocalizing signals had been previously reported in mQTL studies using human osteoarthritic cartilage.[8,11,13,43,44] If the disease effector gene at this locus has been functionally validated, the gene name is shown in parentheses. For functional annotation, we further investigated whether the CpGs fell within or adjacent to (within 150 bp of) narrow peaks in cartilage ATAC-seq datasets (GEO: GSE214394) or within regions enriched for H3K27ac (a marker of enhancer activity) in human limb tissues at embryonic day 47 (Carnegie Stage 19; GEO: GSE42413).

Dimorphism in DNAm levels between the biological sexes has been identified in a multitude of tissues across the human lifespan and is believed to contribute to the discrepancy in disease trajectories between males and females.[56] A recent study demonstrated that developmental sexual dimorphism in DNAm is tissue specific, with a trend for hypomethylation in the placental tissues of female premature neonates, and hypermethylation in blood.[38] Due to the known discrepancies in the prevalence of knee OA in men and women, we investigated sDMRs at autosomal loci to look for sex-specific differences within the developing knee joint. The most significant sDMR mapped to the gene *NAB1*, encoding nerve growth factor induced (NGFI)-A-binding protein 1, a transcriptional repressor[57] that also maps to the most significant DMP identified in neonatal blood. We found no significant interactions in our samples between sex and developmental stage upon DNAm. Sex differences in methylation profiles could potentially be reflective of different subsets of articular chondrocytes between sexes or reflective of established differences in fetal growth patterns and rates, which include femoral length.[58] However, it is equally likely that the observed dimorphism is independent of cellular context, as many of the identified loci have also been identified in neonatal blood[38] and showed no enrichment for processes through GO analysis in either tissue.[36]

Finally, we investigated the presence of epigenome-wide mQTLs within our fetal cartilage samples, discovering ~400,000 significant SNV-CpG associations. Due to the scarcity of the samples used in this study, it was not possible to use a replication cohort to validate our findings. However, comparison of our epigenome-wide mQTLs to those identified in other tissues (fetal brain and adult low-grade OA knee cartilage) identified a proportion of shared effects that was higher in the two cartilage datasets (51%) than between cartilage and brain tissues (29%). Furthermore, shared mQTLs showed high correlations of effect sizes. In 2023, Oliva et al. demonstrated through analysis of mQTLs across nine adult human tissues that mQTLs shared between tissues exhibit larger effect sizes.[59] From a translational perspective, shared effects between tissues are important, especially when comparing tissues such as cartilage (which are relatively difficult to access and biopsy within the joint) to those which are easily obtainable such as blood and saliva.[60] Estimates range between 31% and 68% for shared mQTL effects across tissues, yet the consensus on the use of alternative tissues as a proxy is unclear and would require validation for each tissue type and disease. It has been demonstrated that the correlation coefficient ($\check{r}_b$) between blood and brain mQTLs is high (0.8).[58] A recent mQTL study of adult knee cartilage and whole blood samples also demonstrated that 89% of identified mQTLs in low-grade OA knee cartilage were shared in whole blood samples, yet almost 10% had an opposite direction of effect.[8] When considering trait-specific effects (as opposed to epigenome-wide effects), this additionally does not take tissue-specific gene expression into account, nor chromatin state, which can aid interpretation of functional impacts of such molecular traits.

Colocalization analyses revealed a shared impact of SNVs on fetal cartilage DNAm and OA genetic risk, highlighting trait-specific mQTLs. This confirms and builds upon our earlier report that the genetic and epigenetic interplay underlying OA risk can operate within chondrocytes from the start of life.[21] The majority (67%) of the colocalizations had not previously been identified in studies of aged cartilage, supporting our earlier hypothesis that some epigenetic mechanisms identified in fetal samples may operate throughout the life course, while others may be constrained within the developmental period. We identified that the fetal OA-mQTLs are enriched in chondrocyte enhancers (1.5-fold) and significantly depleted in repressed regions when compared to all significant mQTLs ($p = 0.008$). This supports existing evidence that trait-linked mQTLs primarily impact gene expression through modulation of DNAm within coding (transcribed)[61] regions and distal regulatory elements (as opposed to promoters).[59] Further enrichment analysis also demonstrated that the CpGs stringently regulated by SNVs in a tissue-specific manner from the very start of life are less amenable to modulation by their environment, highlighting their ability to differentially regulate a target gene throughout the life course. While the genes mapping to the mQTL loci remain to be functionally validated, our *in silico* analysis reveals putative regulatory mechanisms.

We identified previously unreported OA-mQTLs mapping to the 3′ UTR (two CpGs) and downstream (one CpG) of *CHST3*, within and adjacent to open chromatin regions. *CHST3* codes for chondroitin-6-*O*-sulfotransferase 1, an enzyme responsible for the sulfation of ECM glycosaminoglycans (GAGs).[62] Sulfation profiles of ECM GAGs within cartilage change throughout development and aging and play a role in cell differentiation and tissue morphogenesis.[62] Mutations in *CHST3* lead to skeletal dysplasia, highlighting the important function of this protein in skeletogenesis.[63] Any conferred changes to the expression of the gene and its encoded protein, mediated through differential methylation, could potentially confer a subtle phenotypic shift within cartilage, altering joint morphology,[64] or decreasing its integrity to withstand mechanical forces over the life course.

We further identified OA-mQTLs in fetal cartilage that map to the genes *ALDH1A2* and *GDF5*, loci at which such effects have previously been identified in aged cartilage. On chromosome 15, an OA-mQTL was identified falling within the first intron of *ALDH1A2*, encoding RALDH2, integral for all-*trans* retinoic acid (atRA) synthesis. The *ALDH1A2* risk locus for hand OA has previously been functionally characterized in adult joint tissues. Allelic expression imbalance at the locus showed a decrease in gene expression driven by the OA risk allele

at rs3204689, which was present in osteochondral tissue from the trapezium, along with cartilage from the hip and knee.[65,66] This effect was also replicated in our targeted analysis of human fetal cartilage.[21] Furthermore, an OA-mQTL was identified in adult low-grade OA cartilage at cg12031962, where the minor and effect allele, C, correlated with a decrease in DNAm.[11] In this study, we colocalized an alternative OA risk SNV at the locus rs11071366 (A>T),[32] which is in moderate pairwise linkage disequilibrium with rs3204689 in European populations ($r^2 = 0.56$). In fetal knee chondrocytes, we also identified an OA-mQTL at the same site, where the effect allele, A, was associated with increased DNAm. Recent studies have shown that atRA has an anti-inflammatory effect within the joint and that pharmacologically blocking its cellular metabolism exhibited a chondroprotective effect.[66] As an example of an epigenetic OA risk mechanism potentially operating throughout the life course, this knowledge could influence the key therapeutic window for future targeted interventions.

In summary, we report a comprehensive analysis of DNA methylation during human skeletal development. Our findings complement existing evidence that genetic factors linked to OA risk exert regulatory influences during the prenatal stage, underscoring the significance of early developmental processes in joint homeostasis and disease in later life. Moreover, our study showcases the effectiveness of mQTL mapping in pinpointing potentially causal regulatory elements associated with common diseases across complex genomic regions. Future studies including both aged and developmental samples could potentially identify subsets of genetic risk loci that may be active across the life course, requiring earlier interventions to prevent the onset and progression of disease. Our data support our current hypothesis of three categories of functional OA risk: (1) loci active only during development; (2) loci active throughout the life course; and (3) loci active only in aged or diseased tissue. When this is known for a locus/pathway, intelligent timing of a pharmacological intervention can then be undertaken, and those which act in later life can be prioritized. Investigations that directly compare multiple joint tissues have also recently proven insightful into understanding the molecular basis of the disease.[8,67] Such studies undertaken throughout the life course will be vital to further elucidate the tissue-specific gene-regulatory networks contributing to disease, paving the way for therapeutic intervention.

Data and code availability

Code used during this project are available on https://github.com/CBFLivUni/Epigenetics-OA-Risk-Human-Skeletal-Dev. Processed DNA methylation data for the 72 samples have been uploaded to the Gene Expression Omnibus. The accession number for the methylation data reported in this paper is GEO: GSE266461.

Acknowledgments

S.J.R. is funded by the Royal Society (RGS\R1\231319), the JGW Patterson Foundation, the MRC-Versus Arthritis Centre for Integrated Research into Musculoskeletal Ageing (CIMA; MR/P020941/1 and MR/R502182/1), and a Versus Arthritis Career Development Fellowship (22615). D.R. is funded by NIH K99AR078352. A.K.S. received research fellowship funding from the Wellcome Trust, Royal College of Surgeons of England, and the UK-US Fulbright commission. S.E.O. is funded through the MRC Discovery Medicine North (DiMeN) Doctoral Training Partnership. The graphical abstract was created in BioRender.

Author contributions

Conceptualization, S.J.R.; methodology, E.M., J.S., A.K.S., and S.J.R.; formal analysis, E.M. and J.S.; investigation, E.M., J.S., S.E.O., M.J.B., A.B., D.R., N.W., L.M., L.M.O., A.K.S., and S.J.R.; resources, N.W., L.M., and L.M.O.; data curation, E.M., J.S., and S.J.R.; writing – original draft, S.J.R.; writing – review & editing, E.M., J.S., D.A.Y., and S.J.R.; visualization, S.E.O., J.S., and S.J.R.; supervision, J.S., S.J.R., and D.A.Y.; project administration, S.J.R.; funding acquisition, S.J.R.

Declaration of interests

L.M. has received speaker and consultancy fees from Illumina.

Supplemental information

Supplemental information can be found online at https://doi.org/10.1016/j.ajhg.2024.10.017.

Received: May 13, 2024
Accepted: October 25, 2024
Published: November 22, 2024

References

1. Finnegan, M.A., and Uhthoff, H.K. (1990). The Development of the Knee. In The Embryology of the Human Locomotor System, pp. 129–140. https://doi.org/10.1007/978-3-642-75310-7_14.
2. Funari, V.A., Day, A., Krakow, D., Cohn, Z.A., Chen, Z., Nelson, S.F., and Cohn, D.H. (2007). Cartilage-selective genes identified in genome-scale analysis of non-cartilage and cartilage gene expression. BMC Genom. *8*, 165. https://doi.org/10.1186/1471-2164-8-165.
3. Geiman, T.M., and Muegge, K. (2010). DNA methylation in early development. Mol. Reprod. Dev. *77*, 105–113. https://doi.org/10.1002/MRD.21118.
4. Cheng, X. (1995). DNA modification by methyltransferases. Curr. Opin. Struct. Biol. *5*, 4–10. https://doi.org/10.1016/0959-440X(95)80003-J.
5. Wolf, S.F., Jolly, D.J., Lunnen, K.D., Friedmann, T., and Migeon, B.R. (1984). Methylation of the hypoxanthine phosphoribosyltransferase locus on the human X chromosome: implications for X-chromosome inactivation. Proc. Natl. Acad. Sci. USA *81*, 2806–2810. https://doi.org/10.1073/PNAS.81.9.2806.

6. Schübeler, D. (2015). Function and information content of DNA methylation. Nature *517*, 321–326. https://doi.org/10.1038/NATURE14192.
7. Roberts, J.B., and Rice, S.J. (2024). Osteoarthritis as an Enhanceropathy: Gene Regulation in Complex Musculoskeletal Disease. Curr. Rheumatol. Rep. *26*, 222–234. https://doi.org/10.1007/S11926-024-01142-Z.
8. Kreitmaier, P., Suderman, M., Southam, L., Coutinho de Almeida, R., Hatzikotoulas, K., Meulenbelt, I., Steinberg, J., Relton, C.L., Wilkinson, J.M., and Zeggini, E. (2022). An epigenome-wide view of osteoarthritis in primary tissues. Am. J. Hum. Genet. *109*, 1255–1271. https://doi.org/10.1016/J.AJHG.2022.05.010.
9. Jeffries, M.A., Donica, M., Baker, L.W., Stevenson, M.E., Annan, A.C., Beth Humphrey, M., James, J.A., and Sawalha, A.H. (2016). Genome-Wide DNA Methylation Study Identifies Significant Epigenomic Changes in Osteoarthritic Subchondral Bone and Similarity to Overlying Cartilage. Arthritis Rheumatol. *68*, 1403–1414. https://doi.org/10.1002/ART.39555.
10. Den Hollander, W., Ramos, Y.F.M., Bos, S.D., Bomer, N., Van Der Breggen, R., Lakenberg, N., De Dijcker, W.J., Duijnisveld, B.J., Slagboom, P.E., Nelissen, R.G.H.H., and Meulenbelt, I. (2014). Knee and hip articular cartilage have distinct epigenomic landscapes: Implications for future cartilage regeneration approaches. Ann. Rheum. Dis. *73*, 2208–2212. https://doi.org/10.1136/ANNRHEUMDIS-2014-205980.
11. Rushton, M.D., Reynard, L.N., Young, D.A., Shepherd, C., Aubourg, G., Gee, F., Darlay, R., Deehan, D., Cordell, H.J., and Loughlin, J. (2015). Methylation quantitative trait locus analysis of osteoarthritis links epigenetics with genetic risk. Hum. Mol. Genet. *24*, 7432–7444. https://doi.org/10.1093/hmg/ddv433.
12. Rice, S.J., Tselepi, M., Sorial, A.K., Aubourg, G., Shepherd, C., Almarza, D., Skelton, A.J., Pangou, I., Deehan, D., Reynard, L.N., and Loughlin, J. (2019). Prioritization of PLEC and GRINA as osteoarthritis risk genes through the identification and characterization of novel methylation quantitative trait loci. Arthritis Rheumatol. *71*, 1285–1296. https://doi.org/10.1002/art.40849.
13. Rice, S.J., Cheung, K., Reynard, L.N., and Loughlin, J. (2019). Discovery and analysis of methylation quantitative trait loci (mQTLs) mapping to novel osteoarthritis genetic risk signals. Osteoarthritis Cartilage *27*, 1545–1556. https://doi.org/10.1016/j.joca.2019.05.017.
14. Rice, S.J., Beier, F., Young, D.A., and Loughlin, J. (2020). Interplay between genetics and epigenetics in osteoarthritis. Nat. Rev. Rheumatol. *16*, 268–281. https://doi.org/10.1038/s41584-020-0407-3.
15. Kehayova, Y.S., Wilkinson, J.M., Rice, S.J., and Loughlin, J. (2023). Osteoarthritis genetic risk acting on the galactosyltransferase gene COLGALT2 has opposing functional effects in articulating joint tissues. Arthritis Res. Ther. *25*, 83. https://doi.org/10.1186/S13075-023-03066-Y.
16. Rice, S.J., Roberts, J.B., Tselepi, M., Brumwell, A., Falk, J., Steven, C., and Loughlin, J. (2021). Genetic and Epigenetic Fine-Tuning of TGFB1 Expression Within the Human Osteoarthritic Joint. Arthritis Rheumatol. *73*, 1866–1877. https://doi.org/10.1002/ART.41736.
17. Pitsillides, A.A., and Beier, F. (2011). Cartilage biology in osteoarthritis - Lessons from developmental biology. Preprint *7*, 654–663. https://doi.org/10.1038/nrrheum.2011.
18. Frysz, M., Faber, B.G., Ebsim, R., Saunders, F.R., Lindner, C., Gregory, J.S., Aspden, R.M., Harvey, N.C., Cootes, T., and Tobias, J.H. (2022). Machine Learning–Derived Acetabular Dysplasia and Cam Morphology Are Features of Severe Hip Osteoarthritis: Findings From UK Biobank. J. Bone Miner. Res. *37*, 1720–1732. https://doi.org/10.1002/jbmr.4649.
19. Faber, B.G., Baird, D., Gregson, C.L., Gregory, J.S., Barr, R.J., Aspden, R.M., Lynch, J., Nevitt, M.C., Lane, N.E., Orwoll, E., et al. (2017). DXA-derived hip shape is related to osteoarthritis: findings from in the MrOS cohort. Osteoarthritis Cartilage *25*, 2031–2038. https://doi.org/10.1016/J.JOCA.2017.09.006.
20. Baird, D.A., Evans, D.S., Kamanu, F.K., Gregory, J.S., Saunders, F.R., Giuraniuc, C.V., Barr, R.J., Aspden, R.M., Jenkins, D., Kiel, D.P., et al. (2019). Identification of Novel Loci Associated With Hip Shape: A Meta-Analysis of Genomewide Association Studies. J. Bone Miner. Res. *34*, 241–251. https://doi.org/10.1002/jbmr.3605.
21. Rice, S.J., Brumwell, A., Falk, J., Kehayova, Y.S., Casement, J., Parker, E., Hofer, I.M.J., Shepherd, C., and Loughlin, J. (2023). Genetic risk of osteoarthritis operates during human skeletogenesis. Hum. Mol. Genet. *32*, 2124–2138. https://doi.org/10.1093/HMG/DDAC251.
22. Li, E., Bestor, T.H., and Jaenisch, R. (1992). Targeted mutation of the DNA methyltransferase gene results in embryonic lethality. Cell *69*, 915–926. https://doi.org/10.1016/0092-8674(92)90611-F.
23. Okano, M., Bell, D.W., Haber, D.A., and Li, E. (1999). DNA methyltransferases Dnmt3a and Dnmt3b are essential for de novo methylation and mammalian development. Cell *99*, 247–257. https://doi.org/10.1016/S0092-8674(00)81656-6.
24. Shen, J., Wang, C., Li, D., Xu, T., Myers, J., Ashton, J.M., Wang, T., Zuscik, M.J., McAlinden, A., and O'Keefe, R.J. (2017). DNA methyltransferase 3b regulates articular cartilage homeostasis by altering metabolism. JCI Insight *2*, e93612. https://doi.org/10.1172/JCI.INSIGHT.93612.
25. Sarkar, A., Liu, N.Q., Magallanes, J., Tassey, J., Lee, S., Shkhyan, R., Lee, Y., Lu, J., Ouyang, Y., Tang, H., et al. (2023). STAT3 promotes a youthful epigenetic state in articular chondrocytes. Aging Cell *22*, e13773. https://doi.org/10.1111/ACEL.13773.
26. Pidsley, R., Zotenko, E., Peters, T.J., Lawrence, M.G., Risbridger, G.P., Molloy, P., Van Djik, S., Muhlhausler, B., Stirzaker, C., and Clark, S.J. (2016). Critical evaluation of the Illumina MethylationEPIC BeadChip microarray for whole-genome DNA methylation profiling. Genome Biol. *17*, 208–217. https://doi.org/10.1186/S13059-016-1066-1.
27. McCartney, D.L., Walker, R.M., Morris, S.W., McIntosh, A.M., Porteous, D.J., and Evans, K.L. (2016). Identification of polymorphic and off-target probe binding sites on the Illumina Infinium MethylationEPIC BeadChip. Genom. Data *9*, 22–24. https://doi.org/10.1016/J.GDATA.2016.05.012.
28. Aryee, M.J., Jaffe, A.E., Corrada-Bravo, H., Ladd-Acosta, C., Feinberg, A.P., Hansen, K.D., and Irizarry, R.A. (2014). Minfi: a flexible and comprehensive Bioconductor package for the analysis of Infinium DNA methylation microarrays. Bioinformatics *30*, 1363–1369. https://doi.org/10.1093/BIOINFORMATICS/BTU049.
29. Roadmap Epigenomics Consortium, Kundaje, A., Meuleman, W., Ernst, J., Bilenky, M., Yen, A., Heravi-Moussavi, A., Kheradpour, P., Zhang, Z., Wang, J., et al. (2015). Integrative analysis

of 111 reference human epigenomes. Nature *518*, 317–330. https://doi.org/10.1038/nature14248.
30. Shabalin, A.A. (2012). Matrix eQTL: ultra fast eQTL analysis via large matrix operations. Bioinformatics *28*, 1353–1358. https://doi.org/10.1093/BIOINFORMATICS/BTS163.
31. Cunningham, F., Allen, J.E., Allen, J., Alvarez-Jarreta, J., Amode, M.R., Armean, I.M., Austine-Orimoloye, O., Azov, A.G., Barnes, I., Bennett, R., et al. (2022). Ensembl 2022. Nucleic Acids Res. *50*, D988–D995. https://doi.org/10.1093/NAR/GKAB1049.
32. Boer, C.G., Hatzikotoulas, K., Southam, L., Stefánsdóttir, L., Zhang, Y., Coutinho de Almeida, R., Wu, T.T., Zheng, J., Hartley, A., Teder-Laving, M., et al. (2021). Deciphering osteoarthritis genetics across 826,690 individuals from 9 populations. Cell *184*, 4784–4818.e17. https://doi.org/10.1016/J.CELL.2021.07.038.
33. Wang, G., Sarkar, A., Carbonetto, P., and Stephens, M. (2020). A Simple New Approach to Variable Selection in Regression, with Application to Genetic Fine Mapping. J. R. Stat. Soc. Series B Stat. Methodol. *82*, 1273–1300. https://doi.org/10.1111/RSSB.12388.
34. Vorontsov, I.E., Eliseeva, I.A., Zinkevich, A., Nikonov, M., Abramov, S., Boytsov, A., Kamenets, V., Kasianova, A., Kolmykov, S., Yevshin, I.S., et al. (2024). HOCOMOCO in 2024: a rebuild of the curated collection of binding models for human and mouse transcription factors. Nucleic Acids Res. *52*, D154–D163. https://doi.org/10.1093/NAR/GKAD1077.
35. Zhang, F., Wang, Y., Zhao, Y., Wang, M., Zhou, B., Zhou, B., and Ge, X. (2023). NFATc1 marks articular cartilage progenitors and negatively determines articular chondrocyte differentiation. Elife *12*, e81569. https://doi.org/10.7554/ELIFE.81569.
36. Carpenter, E.M., Goddard, J.M., Davis, A.P., Nguyen, T.P., and Capecchi, M.R. (1997). Targeted disruption of Hoxd-10 affects mouse hindlimb development. Development *124*, 4505–4514. https://doi.org/10.1242/DEV.124.22.4505.
37. Hecht, J., Stricker, S., Wiecha, U., Stiege, A., Panopoulou, G., Podsiadlowski, L., Poustka, A.J., Dieterich, C., Ehrich, S., Suvorova, J., et al. (2008). Evolution of a core gene network for skeletogenesis in chordates. PLoS Genet. *4*, e1000025. https://doi.org/10.1371/JOURNAL.PGEN.1000025.
38. Santos, H.P., Enggasser, A.E., Clark, J., Roell, K., Zhabotynsky, V., Gower, W.A., Yanni, D., Yang, N.G., Washburn, L., Gogcu, S., et al. (2023). Sexually dimorphic methylation patterns characterize the placenta and blood from extremely preterm newborns. BMC Biol. *21*, 173. https://doi.org/10.1186/S12915-023-01662-7.
39. Richard, D., Capellini, T.D., and Diekman, B.O. (2023). Epigenetics as a mediator of genetic risk in osteoarthritis: role during development, homeostasis, aging, and disease progression. Am. J. Physiol. Cell Physiol. *324*, C1078–C1088. https://doi.org/10.1152/AJPCELL.00574.2022.
40. Capellini, T.D., Chen, H., Cao, J., Doxey, A.C., Kiapour, A.M., Schoor, M., and Kingsley, D.M. (2017). Ancient selection for derived alleles at a GDF5 enhancer influencing human growth and osteoarthritis risk. Nat. Genet. *49*, 1202–1210. https://doi.org/10.1038/ng.3911.
41. Hannon, E., Spiers, H., Viana, J., Pidsley, R., Burrage, J., Murphy, T.M., Troakes, C., Turecki, G., O'Donovan, M.C., Schalkwyk, L.C., et al. (2016). Methylation quantitative trait loci in the developing brain and their enrichment in schizophrenia-associated genomic regions. Nat. Neurosci. *19*, 48–54. https://doi.org/10.1038/NN.4182.
42. Cotney, J., Leng, J., Yin, J., Reilly, S.K., Demare, L.E., Emera, D., Ayoub, A.E., Rakic, P., and Noonan, J.P. (2013). The evolution of lineage-specific regulatory activities in the human embryonic limb. Cell *154*, 185–196. https://doi.org/10.1016/J.CELL.2013.05.056.
43. Aubourg, G., Rice, S.J., Bruce-Wootton, P., and Loughlin, J. (2022). Genetics of osteoarthritis. Osteoarthritis Cartilage *30*, 636–649. https://doi.org/10.1016/j.joca.2021.03.002.
44. Rice, S.J., Tselepi, M., Sorial, A.K., Aubourg, G., Shepherd, C., Almarza, D., Skelton, A.J., Pangou, I., Deehan, D., Reynard, L.N., and Loughlin, J. (2019). Prioritization of PLEC and GRINA as Osteoarthritis Risk Genes Through the Identification and Characterization of Novel Methylation Quantitative Trait Loci. Arthritis Rheumatol. *71*, 1285–1296. https://doi.org/10.1002/art.40849.
45. Chen, H., Capellini, T.D., Schoor, M., Mortlock, D.P., Reddi, A.H., and Kingsley, D.M. (2016). Heads, Shoulders, Elbows, Knees, and Toes: Modular Gdf5 Enhancers Control Different Joints in the Vertebrate Skeleton. PLoS Genet. *12*, e1006454. https://doi.org/10.1371/JOURNAL.PGEN.1006454.
46. Zhang, B., He, P., Lawrence, J.E.G., Wang, S., Tuck, E., Williams, B.A., Roberts, K., Kleshchevnikov, V., Mamanova, L., Bolt, L., et al. (2023). A human embryonic limb cell atlas resolved in space and time. Nature *2023*, 1–11. https://doi.org/10.1038/s41586-023-06806-x.
47. Rux, D., Decker, R.S., Koyama, E., and Pacifici, M. (2019). Joints in the appendicular skeleton: Developmental mechanisms and evolutionary influences. Curr. Top. Dev. Biol. *133*, 119–151. https://doi.org/10.1016/bs.ctdb.2018.11.002.
48. Shwartz, Y., Viukov, S., Krief, S., and Zelzer, E. (2016). Joint Development Involves a Continuous Influx of Gdf5-Positive Cells. Cell Rep. *15*, 2577–2587. https://doi.org/10.1016/j.celrep.2016.05.055.
49. Kim, M., Koyama, E., Saunders, C.M., Querido, W., Pleshko, N., and Pacifici, M. (2022). Synovial joint cavitation initiates with microcavities in interzone and is coupled to skeletal flexion and elongation in developing mouse embryo limbs. Biol. Open *11*, bio059381. https://doi.org/10.1242/bio.059381.
50. Spiers, H., Hannon, E., Schalkwyk, L.C., Smith, R., Wong, C.C.Y., O'Donovan, M.C., Bray, N.J., and Mill, J. (2015). Methylomic trajectories across human fetal brain development. Genome Res. *25*, 338–352. https://doi.org/10.1101/GR.180273.114.
51. Mercader, N., Leonardo, E., Azplazu, N., Serrano, A., Morata, G., Martínez-A, C., and Torres, M. (1999). Conserved regulation of proximodistal limb axis development by Meis1/Hth. Nature *402*, 425–429. https://doi.org/10.1038/46580.
52. Delgado, I., Giovinazzo, G., Temiño, S., Gauthier, Y., Balsalobre, A., Drouin, J., and Torres, M. (2021). Control of mouse limb initiation and antero-posterior patterning by Meis transcription factors. Nat. Commun. *12*, 3086. https://doi.org/10.1038/s41467-021-23373-9.
53. Loomis, C.A., Harris, E., Michaud, J., Wurst, W., Hanks, M., and Joyner, A.L. (1996). The mouse Engrailed-1 gene and ventral limb patterning. Nature *382*, 360–363. https://doi.org/10.1038/382360A0.
54. Lonsdale, J., Thomas, J., Salvatore, M., Phillips, R., Lo, E., Shad, S., Hasz, R., Walters, G., Garcia, F., Young, N., et al. (2013). The Genotype-Tissue Expression (GTEx) project. Nat. Genet. *45*, 580–585. https://doi.org/10.1038/ng.2653.
55. Barter, M.J., Bui, C., Cheung, K., Falk, J., Gómez, R., Skelton, A.J., Elliott, H.R., Reynard, L.N., and Young, D.A. (2020).

DNA hypomethylation during MSC chondrogenesis occurs predominantly at enhancer regions. Sci. Rep. *10*, 1169. https://doi.org/10.1038/S41598-020-58093-5.

56. Martin, E., Smeester, L., Bommarito, P.A., Grace, M.R., Boggess, K., Kuban, K., Karagas, M.R., Marsit, C.J., O'Shea, T.M., and Fry, R.C. (2017). Sexual epigenetic dimorphism in the human placenta: implications for susceptibility during the prenatal period. Epigenomics *9*, 267–278. https://doi.org/10.2217/EPI-2016-0132.
57. Thiel, G., Kaufmann, K., Magin, A., Lietz, M., Bach, K., and Cramer, M. (2000). The human transcriptional repressor protein NAB1: expression and biological activity. Biochim. Biophys. Acta *1493*, 289–301. https://doi.org/10.1016/S0167-4781(00)00207-4.
58. Qi, T., Wu, Y., Zeng, J., Zhang, F., Xue, A., Jiang, L., Zhu, Z., Kemper, K., Yengo, L., Zheng, Z., et al. (2018). Identifying gene targets for brain-related traits using transcriptomic and methylomic data from blood. Nat. Commun. *9*, 2282. https://doi.org/10.1038/S41467-018-04558-1.
59. Oliva, M., Demanelis, K., Lu, Y., Chernoff, M., Jasmine, F., Ahsan, H., Kibriya, M.G., Chen, L.S., and Pierce, B.L. (2022). DNA methylation QTL mapping across diverse human tissues provides molecular links between genetic variation and complex traits. Nat. Genet. *55*, 112–122. https://doi.org/10.1038/s41588-022-01248-z.
60. Villicaña, S., and Bell, J.T. (2021). Genetic impacts on DNA methylation: research findings and future perspectives. Genome Biol. *22*, 1–35. https://doi.org/10.1186/S13059-021-02347-6.
61. Min, J.L., Hemani, G., Hannon, E., Dekkers, K.F., Castillo-Fernandez, J., Luijk, R., Carnero-Montoro, E., Lawson, D.J., Burrows, K., Suderman, M., et al. (2021). Genomic and phenotypic insights from an atlas of genetic effects on DNA methylation. Nat. Genet. *53*, 1311–1321. https://doi.org/10.1038/S41588-021-00923-X.
62. Kitagawa, H., Tsutsumi, K., Tone, Y., and Sugahara, K. (1997). Developmental regulation of the sulfation profile of chondroitin sulfate chains in the chicken embryo brain. J. Biol. Chem. *272*, 31377–31381. https://doi.org/10.1074/JBC.272.50.31377.
63. Thiele, H., Sakano, M., Kitagawa, H., Sugahara, K., Rajab, A., Höhne, W., Ritter, H., Leschik, G., Nürnberg, P., and Mundlos, S. (2004). Loss of chondroitin 6-O-sulfotransferase-1 function results in severe human chondrodysplasia with progressive spinal involvement. Proc. Natl. Acad. Sci. USA *101*, 10155–10160. https://doi.org/10.1073/PNAS.0400334101.
64. Richard, D., Liu, Z., Cao, J., Kiapour, A.M., Willen, J., Yarlagadda, S., Jagoda, E., Kolachalama, V.B., Sieker, J.T., Chang, G.H., et al. (2020). Evolutionary Selection and Constraint on Human Knee Chondrocyte Regulation Impacts Osteoarthritis Risk. Cell *181*, 362–381.e28. https://doi.org/10.1016/j.cell.2020.02.057.
65. Shepherd, C., Zhu, D., Skelton, A.J., Combe, J., Threadgold, H., Zhu, L., Vincent, T.L., Stuart, P., Reynard, L.N., and Loughlin, J. (2018). Functional Characterization of the Osteoarthritis Genetic Risk Residing at ALDH1A2 Identifies rs12915901 as a Key Target Variant. Arthritis Rheumatol. *70*, 1577–1587. https://doi.org/10.1002/art.40545.
66. Zhu, L., Kamalathevan, P., Koneva, L.A., Zarebska, J.M., Chanalaris, A., Ismail, H., Wiberg, A., Ng, M., Muhammad, H., Walsby-Tickle, J., et al. (2022). Variants in ALDH1A2 reveal an anti-inflammatory role for retinoic acid and a new class of disease-modifying drugs in osteoarthritis. Sci. Transl. Med. *14*, eabm4054. https://doi.org/10.1126/SCITRANSLMED.ABM4054.
67. Kreitmaier, P., Park, Y.-C., Swift, D., Gilly, A., Wilkinson, J.M., Zeggini, E., and Wilkinson, † J.M. (2024). Epigenomic profiling of the infrapatellar fat pad in osteoarthritis. Hum. Mol. Genet. *33*, 501–509. https://doi.org/10.1093/HMG/DDAD198.

The American Journal of Human Genetics, Volume *111*

Supplemental information

The methylomic landscape of human articular cartilage development contains epigenetic signatures of osteoarthritis risk

Euan McDonnell, Sarah E. Orr, Matthew J. Barter, Danielle Rux, Abby Brumwell, Nicola Wrobel, Lee Murphy, Lynne M. Overman, Antony K. Sorial, David A. Young, Jamie Soul, and Sarah J. Rice

Supplemental Data

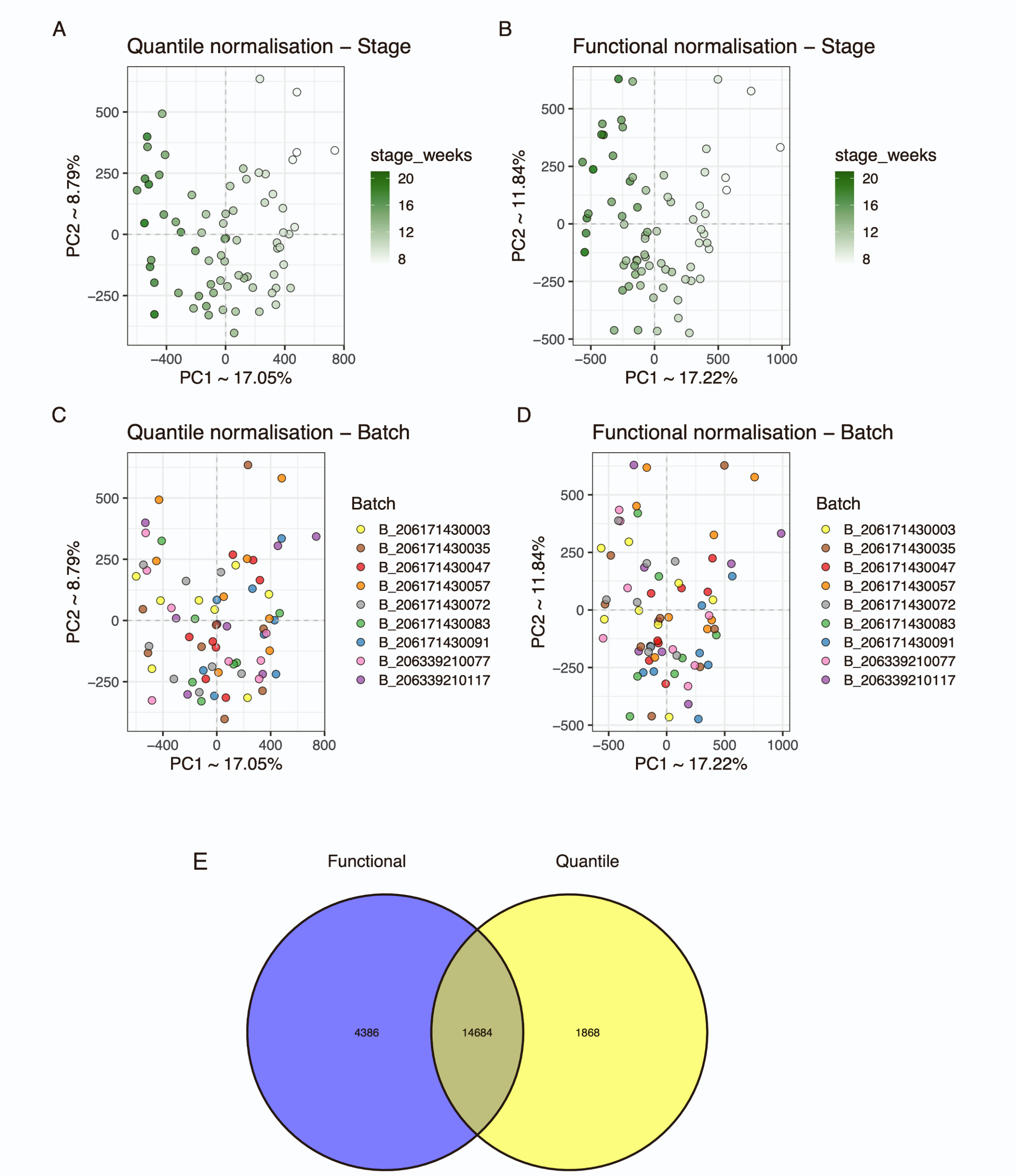

Supplemental Figure 1. Sensitivity of principal component analysis to normalisation approach. **A-D**, Principal component analysis of quantile (A, C) or functional (B, D) normalised methylation data coloured by sample developmental stage or Sentrix ID (batch) shows limited effect of normalisation choice. **E**, Venn diagram of the significant Bonferroni corrected developmental differential methylated probes shows high overlap of differentially methylated probes with quantile and functional normalisation.

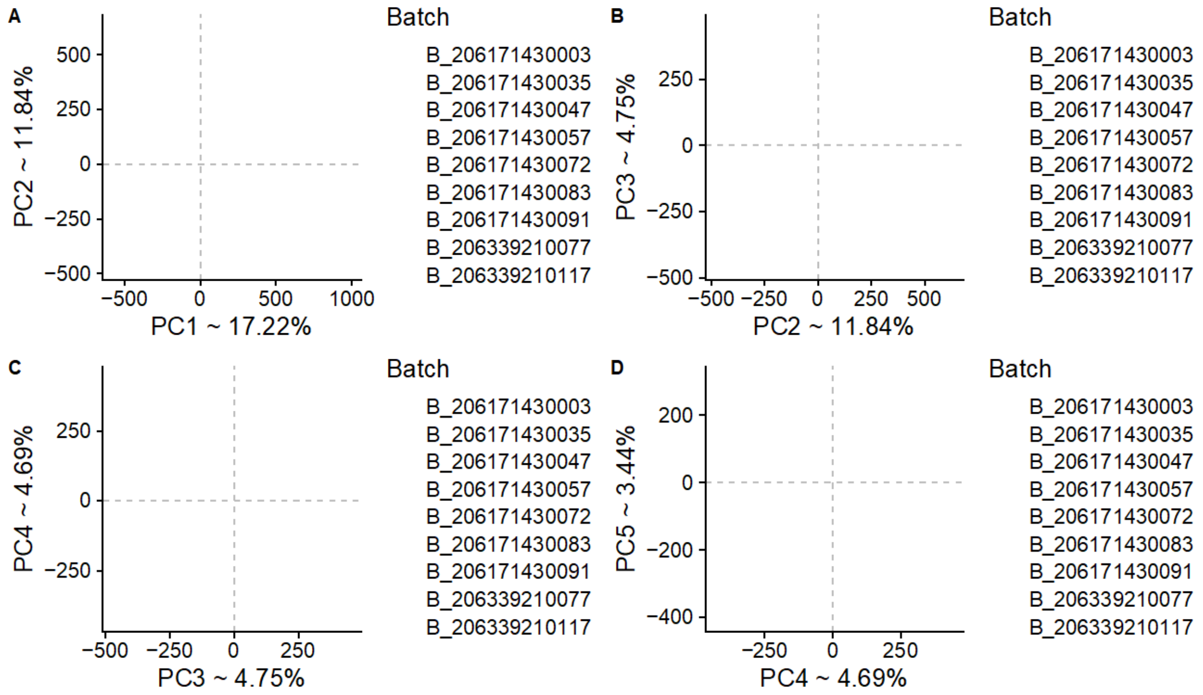

Supplemental Figure 2. Assessment of batch effects by principal component analysis of the 71 foetal cartilage samples used in this study. Principal component analysis of normalised DNAm samples coloured by Sentrix ID shows evidence of weak batch effects, observable in association with PC4.

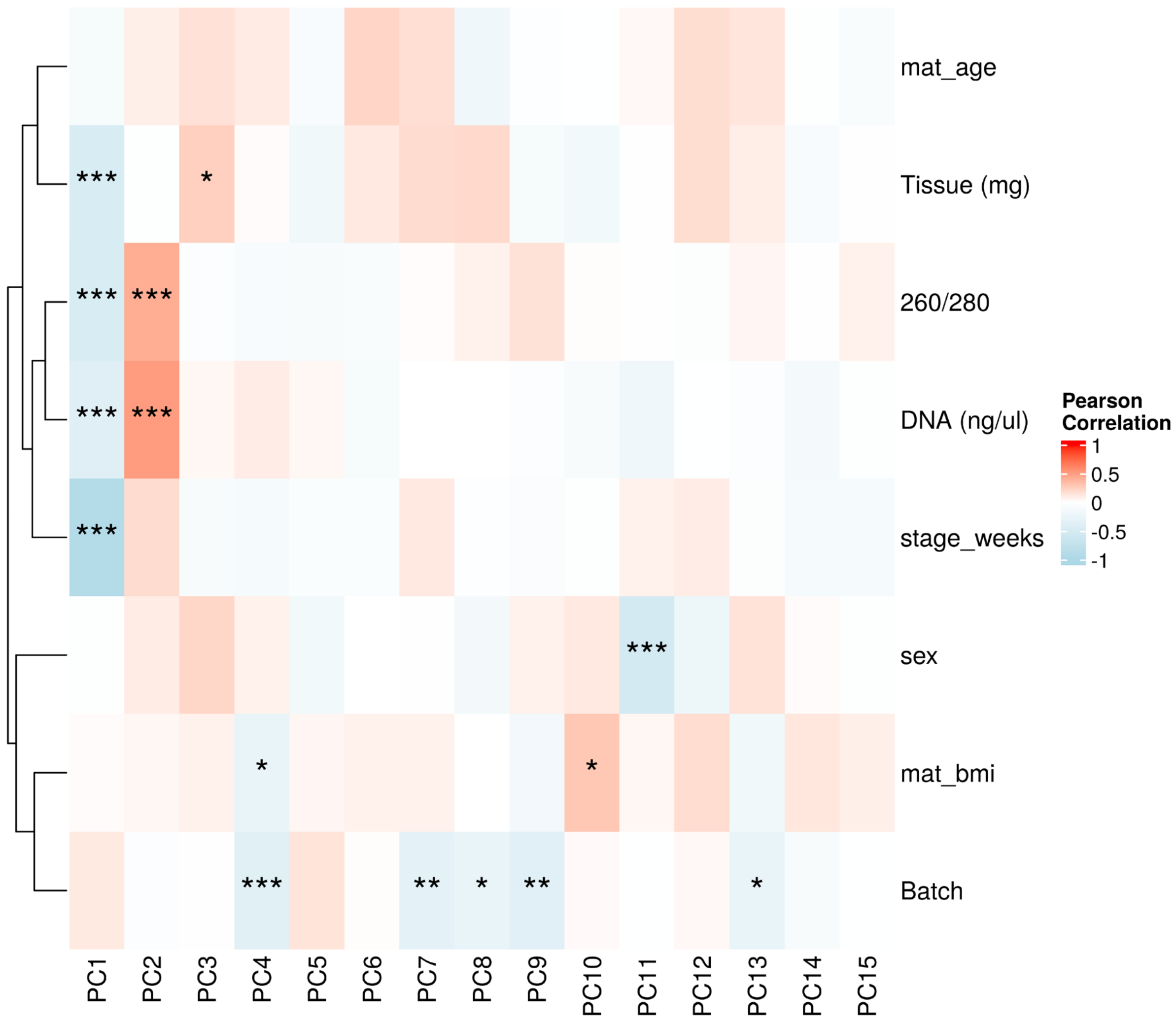

Supplemental Figure 3. Heatmap of Pearson's correlations between calculated methylation principal components and known biological and technical covariates. * p-value < 0.05, ** p-value < 0.01, *** p-value < 0.001

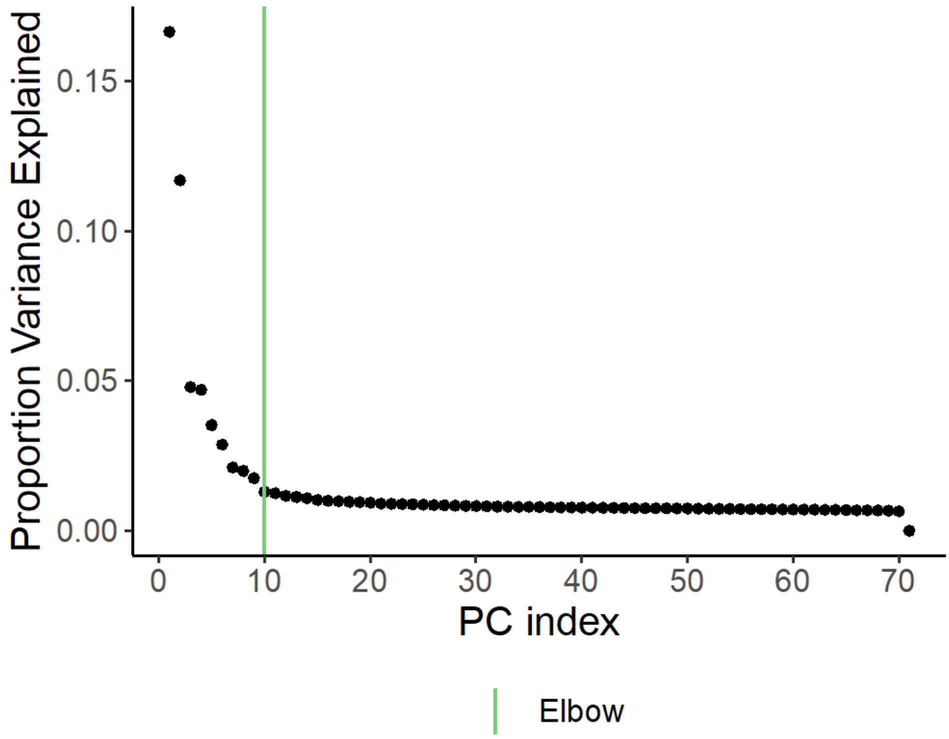

Supplemental Figure 4. Selection of methylation principal components for mQTL analysis. Principal component analysis was performed on the methylation M-values. The variance explained by each principal component is shown and 10 principal components were selected for inclusion in the mQTL analysis using the elbow method of PCAForQTL.

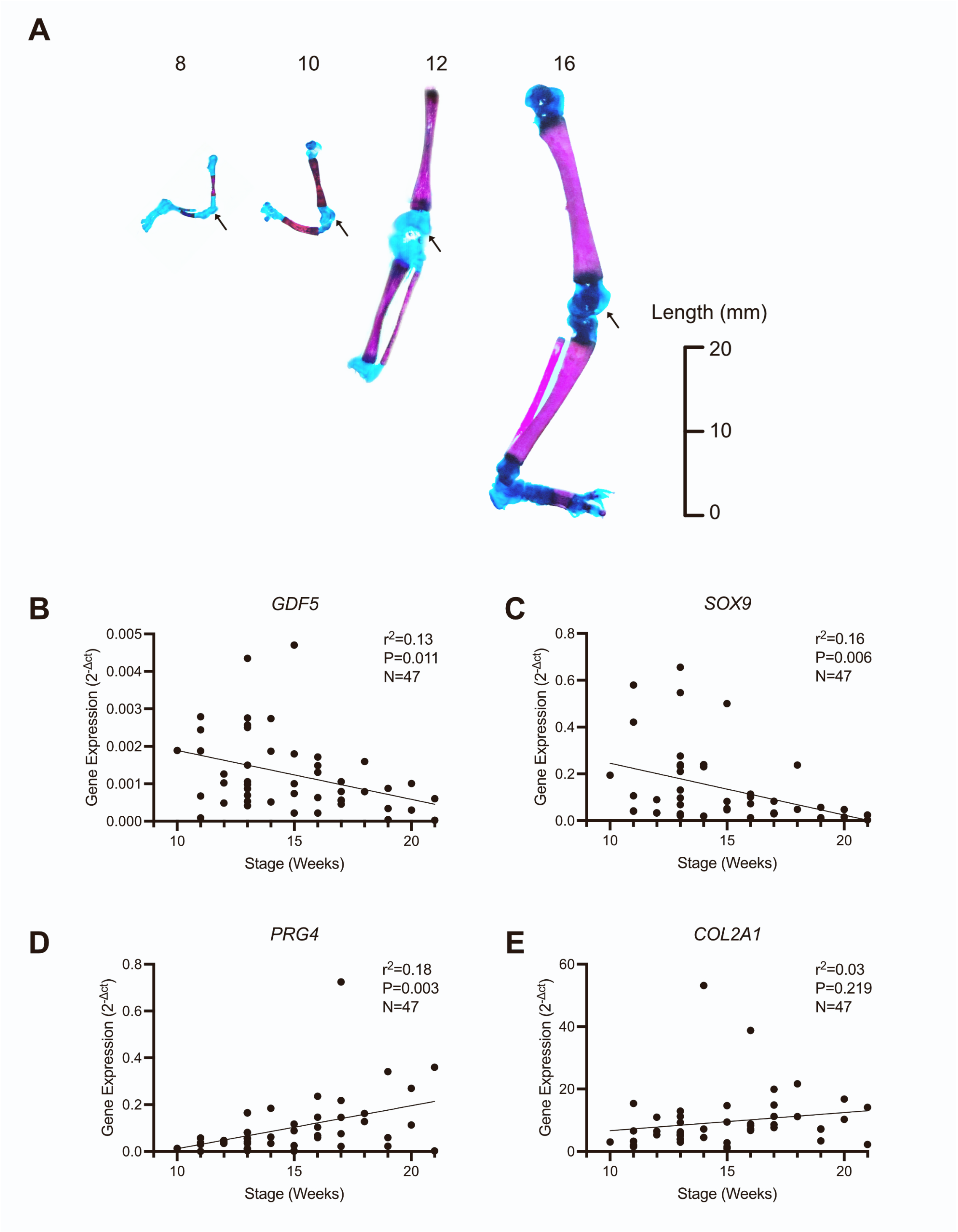

Supplemental Figure 5. Characterisation of the developing human knee cartilage samples. A, Alcian blue (cartilage) and alizarin red (bone) staining of human foetal femur at (L-R) 8, 10, 12, and 16 pcw. Arrows indicate the lower femoral articular surface **B-E,** Gene expression of cartilage progenitor (*GDF5* and *SOX9*) and chondrocyte (*PRG4* and *COL2A1*) makers in the samples. Simple linear regression was performed by the developmental stage.

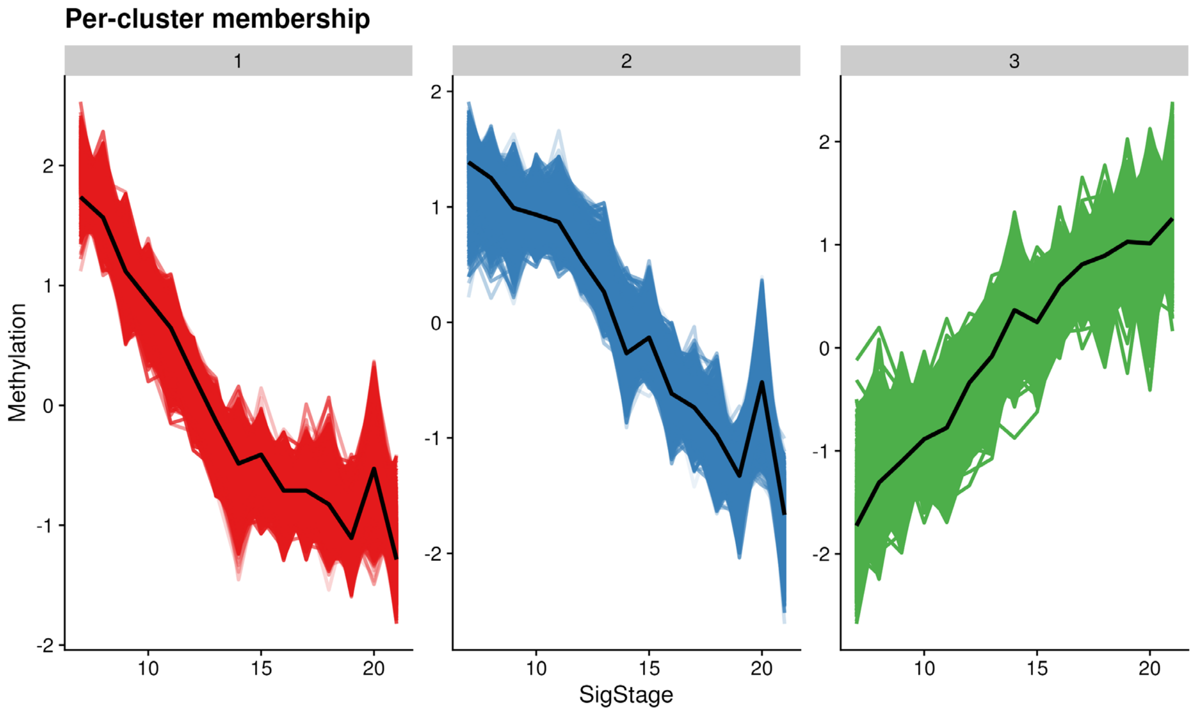

Supplemental Figure 6. The identified dDMPs fell into three distinct clusters based upon the methylation trends across the developmental window. The mfuzz plot for each cluster is displayed. Cluster 1, red; Cluster 2, blue; Cluster 3, green. Methylation is plotted using M-values.

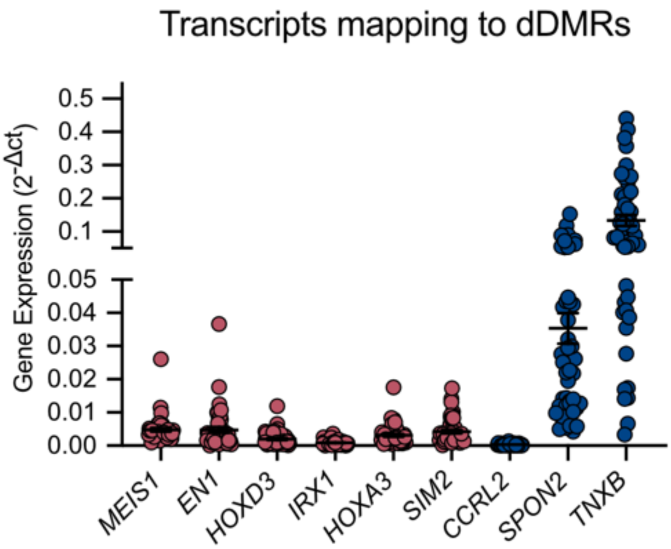

Supplemental Figure 7. Expression of genes mapping to the top hypermethylated (red) and hypomethylated (blue) developmental DMRs (dDMRs).

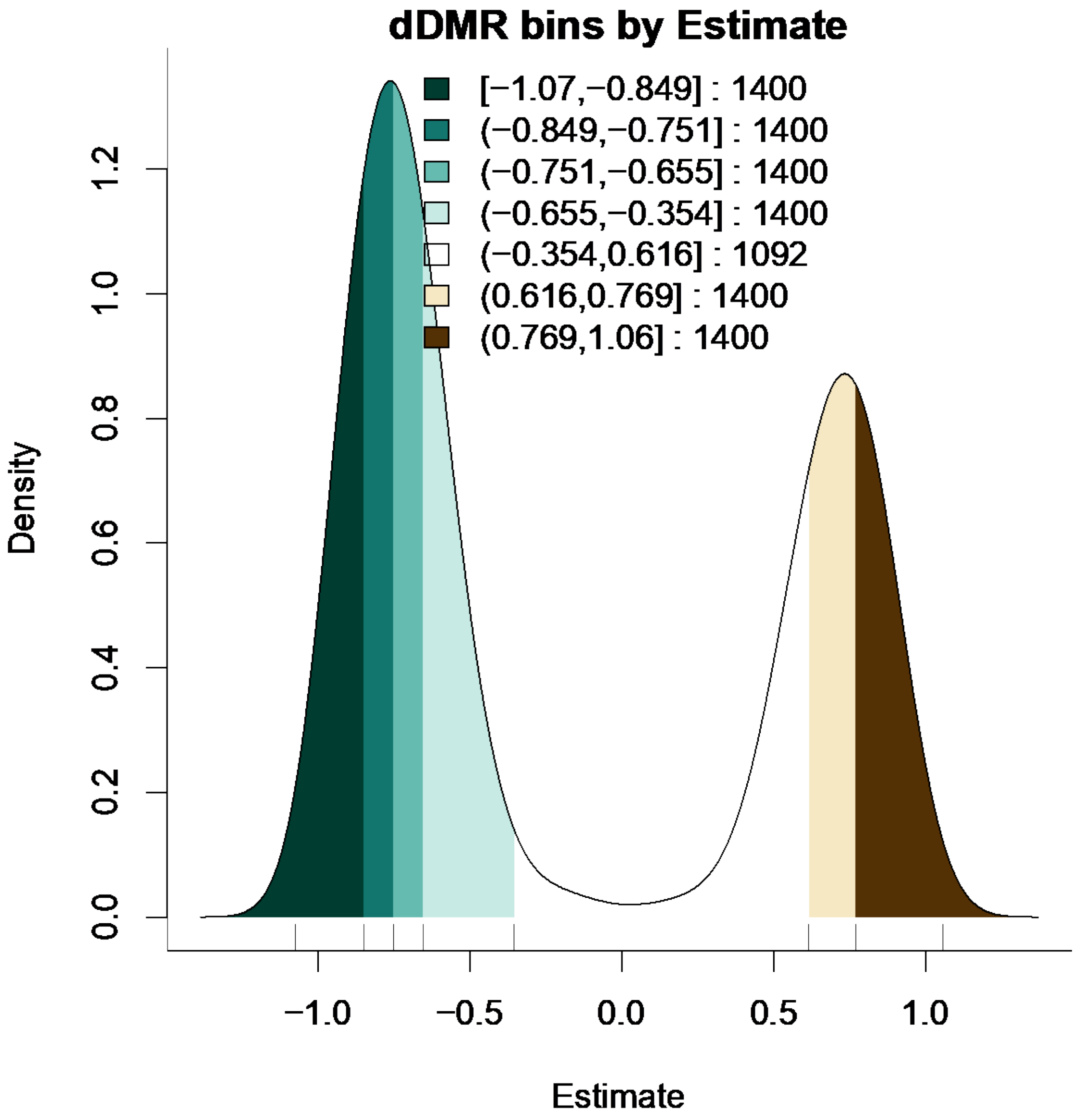

Supplemental Figure 8. Developmental differentially methylated region (dDMR) bins for transcription factor motif analysis. Histogram of the equally sized groups of developmental DMRs binned by Estimate (log2 fold change) that were used for transcription factor motif analysis with monaLisa.

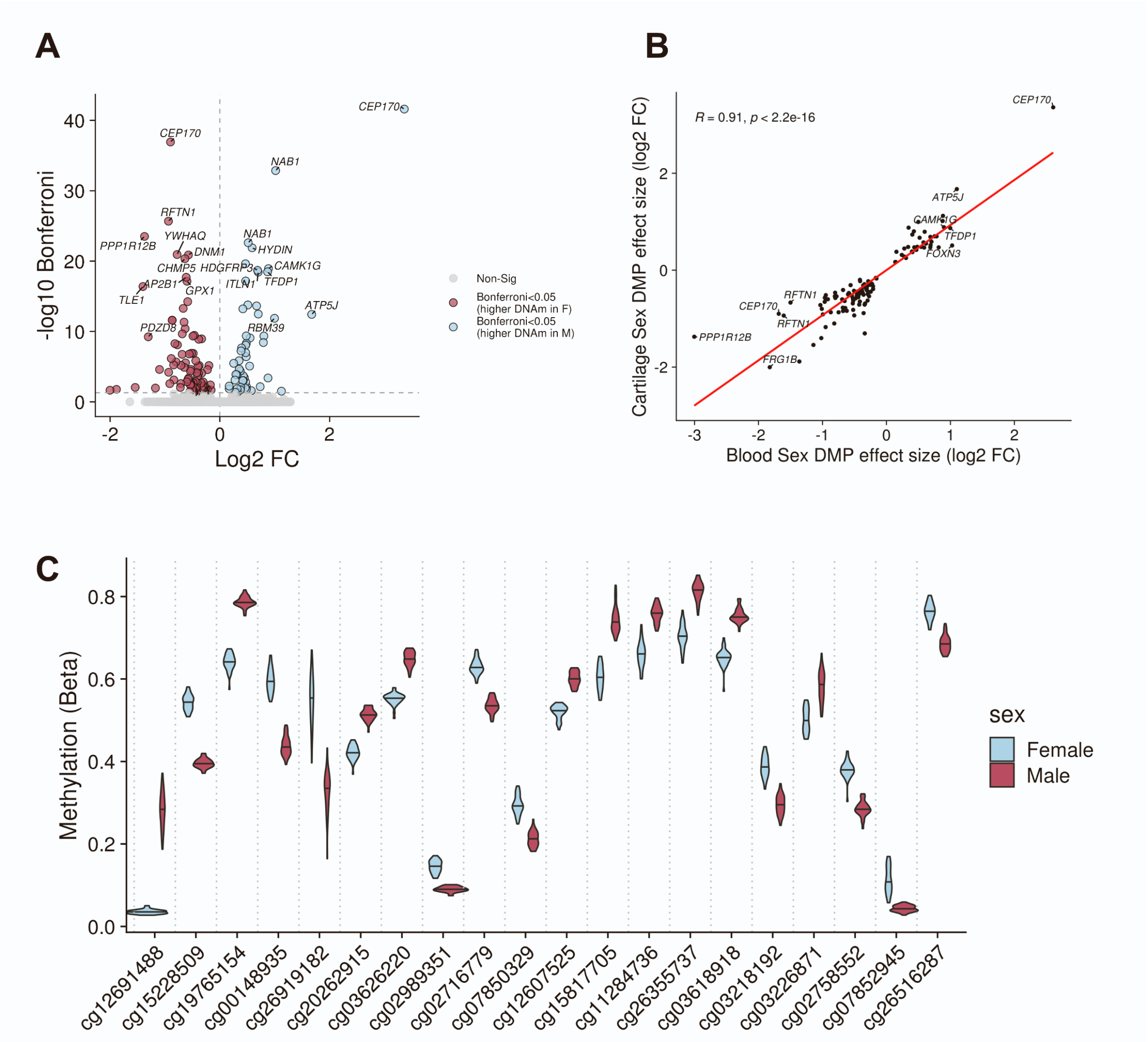

Supplemental Figure 9. Sexual dimorphism in DNA methylation within human developmental cartilage. A, Volcano plot of sDMPs. Grey, non-significant sDMPs (Bonferroni <0.05); Blue, significant sDMPs with higher methylation in male (M) samples (Bonferroni < 0.05); Red, significant sDMPs with higher methylation in female (F) samples (Bonferroni < 0.05); B, Comparison of effect sizes (log2 fold change) for overlapping significant (Bonferroni < 0.05) sDMPs in neonatal blood reported in Santos et al. and foetal cartilage. Genes nearest to the top hyper- and hypomethylated probes are highlighted; D, Violin plot of methylation beta values in human foetal cartilage samples at the most significant sDMP CpGs. Red, male; Blue, female.

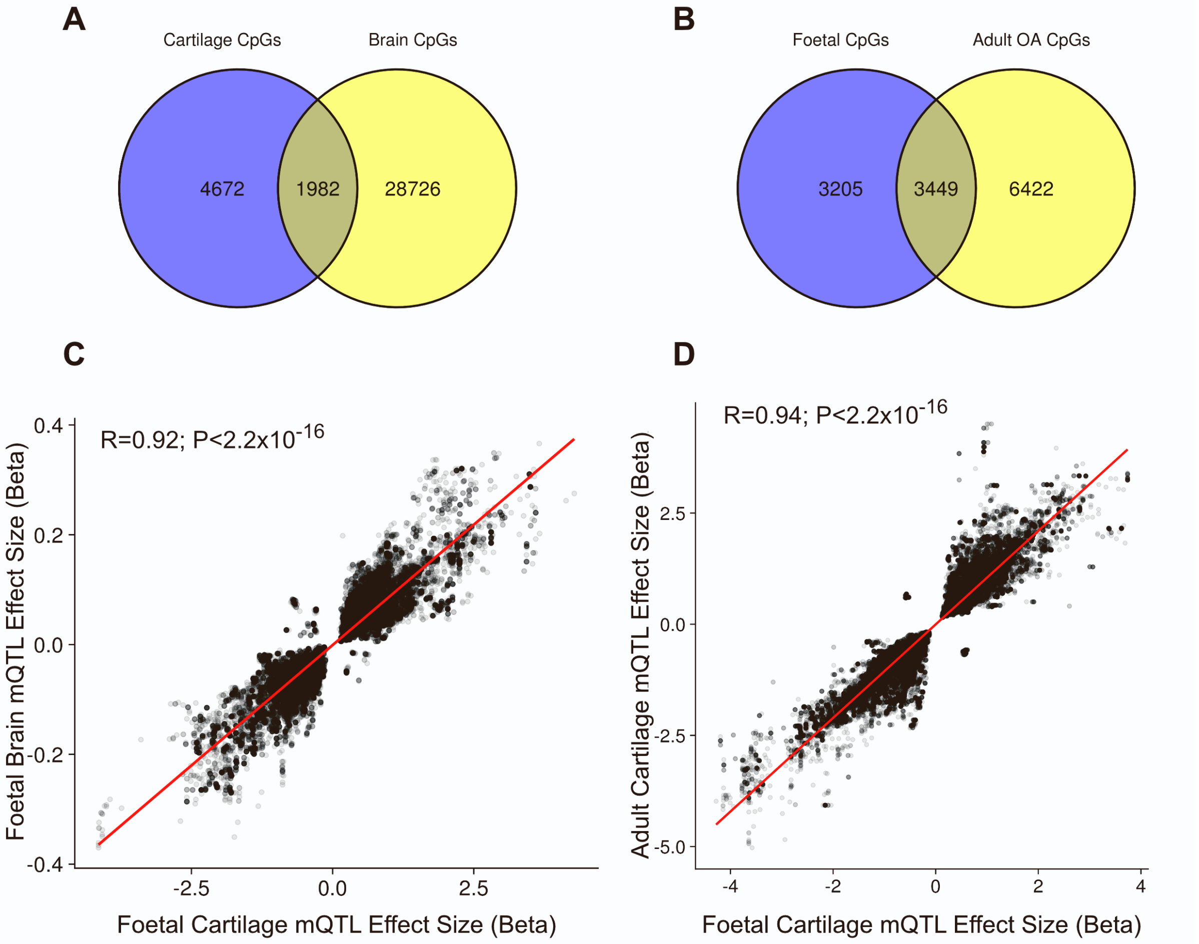

Supplemental Figure 10. Comparison of mQTLs with existing foetal brain and adult osteoarthritic cartilage datasets. A, Venn diagram of the overlapping significant CpGs involved in mQTLs in foetal brain and foetal cartilage mQTLs; B, Venn diagram of the overlapping significant CpGs involved in mQTLs in adult low grade (macroscopically intact) osteoarthritic knee cartilage and foetal cartilage mQTLs; C, Comparison of effect sizes (beta) between the shared significant mQTLs in foetal brain and foetal cartilage mQTLs; D, Comparison of effect sizes (beta) between the shared significant mQTLs in adult cartilage and foetal cartilage mQTLs**.**

Sample information

The developmental maturity of the embryonic samples (<10 gestation weeks) are staged according to the Carnegie staging classification system, which are determined using the HDBR staging guide (https://hdbratlas.org/staging-criteria/carnegie-staging.html) which itself is based upon the staging guide presented in Bullen and Wilson (1997)[1]. Fetal samples are staged from the time of conception, which is estimated by HDBR during sample collection (https://hdbratlas.org/staging-criteria/fetal-staging.html) based on the methods of Hern[2]. All of the data presented here considers the development of the embryo using Carnegie stage (CS) and fetus in post-conception weeks (pcw). Detailed information about the sample characteristics is included in Table S1, along with available maternal data.

Sample isolation and nucleic acid extraction

Nascent articular and epiphyseal cartilage tissue (20-30mg) was taken from the distal end of the developing femur. As anticipated, adipose tissue was visible within the joint from ~19pcw and was thoroughly removed before cartilage isolation. In all cases, tissue was homogenized in 2ml screw-cap tubes containing 600μl RTL lysis buffer (AllPrep, Qiagen) at 2400rpm for 2.5min in 2mm Zirconia beads using the Mini BeadBeater 24 (both from BioSpec Products, USA). Samples were centrifuged at 13,000rpm for 30s before further homogenizing at 2400rpm for 5min. Samples were centrifuged again before clarification through 0.2μM nylon filters (GeneFlow, UK). DNA was isolated using AllPrep (Qiagen) and RNA was isolated using Norgen DNA/RNA mini purification kit (Norgen BioTek Corp, Canada).

Staining of bone and cartilage tissues

Fetal samples were stained following a previously published protocol[3], adapted for human tissue. Briefly, excess tissue was removed from the limb before fixing in 70% ethanol for 24hrs, then 95% ethanol for 24hrs. Ethanol was replaced with 0.03% Alcian blue solution for 1-3 days, depending on fetal stage, then washed with 95% ethanol for 6 hours, before replacement with 2% KOH solution for 12-24 hours. The KOH was changed to 0.005% Alizarin red for 12-

24 hours, before clearance in 1%KOH:20% glycerol solution for 1-5 days. All stages took place with gentle agitation. The samples were imaged using brightfield optics.

DNA methylation quality control

The minfi[4] R package (v1.4.6) was used to read in, process and perform quality control checks on methylation microarray data[5]. Bisulfite conversion and sex prediction checks were performed, and one sample was excluded from the analysis due to a mismatch between predicted and labelled sex. Non-autosomal sites were then excluded. Next, undetected probes (sum of Beta values across all samples equal to 0) and probes detected in less than 3 replicate beads were removed. Probes were then filtered to retain those with a detection P-value <0.001 and any probes with missing data (marked as not applicable, NA) in any samples were excluded. Functional normalization was then applied to the microarray data. Probes covered by known single nucleotide variants (SNVs) and cross-reactive probes were removed using the maxprobes R package (v0.02)[5]. After quality control 678,267 probes representing CpG sites were retained for analysis.

Potential outlier probes were highlighted using the Tukey method implemented in the WateRMelon (v.2.4.0)[6] function pwod with default parameters. Outlier probes were flagged in the downstream DMP and mQTL results, but not removed or masked from analysis due to their observed association with developmental stage.

DNA methylation exploratory data analysis

Methylation data were transformed to M-values and filtered to remove the bottom 5% of probes by variance. Principal component analysis (PCA) was performed using the factoExtra R package (v1.0.7), using Z-scaled data as input (Figure S1). This PCA analysis is hereby referred to as the "methylation PCA" and associated principal components (PC) are "methylation PCs". Visual assessment of batch effect on these PCA plots using Sentrix ID

indicated methylation PC4 was associated with batch (Figure S2). Sentrix ID was therefore incorporated as a covariate in downstream statistical models. To formally determine if known biological or technical covariates were associated with variance in the methylation data, we correlated (Pearson's r) methylation PC1-15 with stage, maternal age, maternal BMI, and sex variables (biological variables) and tissue mass (mg), A260/A280 ratio, DNA concentration (ng/µl) and batch variables (technical variables) (Figure S3). To investigate the presence of any unknown technical factors, we performed n.sva using the Leek method with a model that included the known methylation array IDs. No significant surrogate variables were detected, indicating no major unknown sources of technical variation.

Differential methylation and differential methylated region analyses

Prior to statistical analyses, all data were transformed to M values. Probe-level differential methylation analysis was performed using the limma R package[7] (v3.55.5), using both the standard hypothesis testing method (null hypothesis of log2FC equal to 0) and the Testing Relative to A Threshold (treat) hypothesis testing approach (null hypothesis of log2FC equal to or less than 0.1)[8]. This treat method was taken forward for stage-associated differential methylation analysis, while the standard hypothesis testing method was used for sex-associated differential methylation. Limma linear models for methylation analysis included maternal BMI, maternal age and developmental stage as Z-scaled covariates, with sex as a binary factor. Coefficients and p-values were calculated with developmental stage and sex for developmental- and sex-associated CpGs, respectively. Reference-based analysis was not performed to adjust for variable cell populations in samples due to the homogeneity of cartilage tissue (single cell type, chondrocytes). Post-model fitting and testing, sex- and stage-DMPs were defined by filtration at a Bonferroni P-value of 0.05.

We investigated potential enrichment for up or down regulated DMPs in imprinted genes (obtained from geneimprint.com). We utilized gometh of the MissMethyl package[9] to test for

significant enrichment. No statistically significant enrichment was observed (P>0.05).

Differential methylated region (DMR) analysis with respect to the developmental stage was performed using the dmrff R package (v1.1.0) on the output p-values and standard errors of the probe-level differential methylation analysis. Probes were tested for association into a DMR with a maximum gap between probes of 1000 bp and DMRs defined as containing at least 2 probes and comprising the region between the 5' and 3'-most constituent probes. Resultant candidate sex DMRs (sDMRs) and developmental DMRs (dDMRs) were filtered by an Bonferroni of 0.05.

Sensitivity to Normalization Approach

To examine the sensitivity of the DMP analysis to the choice of methylation normalization, we also performed both quantile and functional normalization. The resulting PCAs showed close similarity after both modes of normalization and high overlap of differentially methylated probes (Figure S1). Functional normalization, that uses control probes as part of the normalization, was selected for use throughout the main analysis due to a higher percentage variance explained by PC2 and to minimize the potential for any confounding technical variables.

DMP clustering with mFuzz

M values for significant stage DMPs were clustered using the Mfuzz R package[10] (v2.62.0) with the optimal cluster number chosen as 3, based on the elbow method. Minimum cluster membership was chosen as 0.99 with a seed value of 5 and the "fuzzifier" parameter was calculated as 1.15 using the "mestimate" function of Mfuzz.

DMR overlap analysis

DMR regions were overlapped within the bounds of Assay for Transposase Accessible Chromatin (ATAC)-Seq peaks previously generated in 12pcw foetal knee (distal femoral)

cartilage[11] and the 15 chromatin-state model predictions for human cultured chondrocytes (E049) downloaded from the NIH ROADMAP Epigenomics Mapping Consortium[12]. Prior to overlap, the 15 ROADMAP states were collapsed into 5 functional classifications: enhancer (Enh, EnhG, EnhBiv), repressed (ZNF/Rpts, Quies, Het, ReprPC, ReprPCWk), transcription start site (TssBiv, TssA), flanking transcription start site (TssAFlnk, BivFlnk) and transcribed (TxWk, Tx, TxFlnk). Regions were overlapped using the intersect function in the bedtools (v2.31.1) package[13]. Presented frequencies and proportions represent overlaps between DMRs and ATAC regions/ROADMAP state classes, not the frequencies of unique members of either of these features. State enrichments were performed using two-sided Fisher's exact tests, testing for frequencies of overlaps with hypo- or hypermethylated DMRs.

GO Meth term enrichment analysis.

Gene ontology (GO) term enrichment analysis was performed on MFuzz clusters and sex DMPs using the gometh of the MissMethyl package[9] (v1.36.0), while DMR enrichment analysis made use of the goregion function. The Gene Ontology (GO) database was used with array.type="EPIC" argument provided to provide DMP-gene annotations. All 678,267 CpGs detected above thresholds were provided as background for all comparisons. Terms were called as significant with an FDR threshold < 0.05.

TF Motif Enrichment Analysis

TF Motif PWMs were downloaded from HOCOMOCO v12[14] and filtered to retain motifs with a quality grade of C and above. MonaLisa (v1.9.0) was used to group all identified dDMRs into bins of 1400 DMRs by fold change[15]. To allow comparison between bins, the DMR sequences were trimmed to the median DMR size (253bp), centered around the center of each DMR (Figure S9). Enrichment statistics (Fisher's exact test) were calculated by comparing occurrences of each motif in a bin to its occurrences in all other bins. Hierarchical clustering of the motif similarity of the enriched motifs was used to visualize the enrichment scores.

Genotype calling and genotype data filtration and quality control

Genotypes were called from raw idat files using gencall and associated array cluster and manifest files. The resultant gtc files were converted to vcf via the gtc_to_vcf.py script of the GTCtoVCF codebase (https://github.com/Illumina/GTCtoVCF). PLINK (v1.90b6.21) was used for subsequent analytic steps, except where otherwise stated[16]. Strand-flipped SNVs were identified by performing sample-wise merges with all remaining samples before all sample data was merged to one PLINK dataset. Genotype data filtering was performed using a minor allele frequency (MAF) > 0.01, while >0.975 was used as the variant and individual genotyping rate filtration threshold. X chromosome pseudoautosomal regions (PARs) were split to a separate chromosome, "26". Sex and interrelatedness checks were performed using PLINK.

Imputation

Pre-imputation checks made use of the checkVCF utility (https://github.com/zhanxw/checkVCF/tree/master). All samples were submitted for imputation, while retaining only autosomes within each sample. Imputation was performed via submission to the Michigan Imputation Server (https://imputationserver.sph.umich.edu/) with population set to "EUR", genome build as "hg19" and using the haplotype reference consortium (HRC) as reference population[17,18]. Imputed vcf files were then filtered to split multiallelic sites into separate records and sites filtered by $r^2>0.3$. Imputed variants were then filtered with MAF > 0.05, alongside >0.975 for both variant and individual genotyping rates. Pre-imputation and post-imputation processing made use of bcftools (v1.16) and post-imputation checks utilised the ic utility (v1.0.9) of the batch McCarthy Group Tools suite[19]. After imputation and filtering, 5,394,299 variants were used in the subsequent mQTL analysis.

1000 genomes project PCA

PLINK2 format data (pgen, pvar and psam files) for the 1000 genomes project were converted to PLINK format data (.bed, .bim and .fam) and these were used as the reference population data[20]. For both reference and study data, A-T and C-G alleles were excluded and multiallelic

sites were entirely excluded, using the "--biallelic strict" option. Next, sites of high linkage-disequilibrium (LD) were removed from the study data using a database of sites provided by the R package plinkQC (v0.3.4). Sites in the study data were then filtered with a 50kb sliding window, moving in 5kb steps and removing sites with a pairwise $r^2>0.2$. Sites in the reference data were then filtered by this pruned list of study data variants. Mismatched chromosome and position IDs were then identified and rectified, and variants orientated on the opposite strands between reference and study data were flipped, followed by the merging of the study and reference datasets. PCA was then performed using PLINK to obtain ancestry related principal components for inclusion in mQTL analysis.

Comparison to mQTL datasets

Fetal brain and adult low grade cartilage mQTLs were downloaded from http://epigenetics.essex.ac.uk/mQTL//All_Imputed_BonfSignificant_mQTLs.csv.gz and https://personal.broadinstitute.org/ryank/Kreitmaier_2022_mqtl_lgcart.txt.gz. For comparison between datasets the Bonferroni significant mQTLs and those CpGs present on the Illumina 450K array were retained. For effect size comparison mQTLs were normalized to the same reference allele and Pearson correlation performed.

Supplemental References

1. Strachan, T., Lindsay, S. (Susan), and Wilson, D.I. (David I.) (1997). Molecular genetics of early human development. 265.
2. Hern, W.M. (1984). Correlation of fetal age and measurements between 10 and 26 weeks of gestation. Obstetrics and Gynecology *63*, 26–32.
3. Rigueur, D., and Lyons, K.M. (2014). Whole-mount skeletal staining. Methods Mol Biol *1130*, 113–121. https://doi.org/10.1007/978-1-62703-989-5_9.
4. Aryee, M.J., Jaffe, A.E., Corrada-Bravo, H., Ladd-Acosta, C., Feinberg, A.P., Hansen, K.D., and Irizarry, R.A. (2014). Minfi: a flexible and comprehensive Bioconductor package for the analysis of Infinium DNA methylation microarrays. Bioinformatics *30*, 1363–1369. https://doi.org/10.1093/BIOINFORMATICS/BTU049.
5. Aryee, M.J., Jaffe, A.E., Corrada-Bravo, H., Ladd-Acosta, C., Feinberg, A.P., Hansen, K.D., and Irizarry, R.A. (2014). Minfi: a flexible and comprehensive Bioconductor package for the analysis of Infinium DNA methylation

microarrays. Bioinformatics *30*, 1363–1369. https://doi.org/10.1093/BIOINFORMATICS/BTU049.
6. Pidsley, R., Y Wong, C.C., Volta, M., Lunnon, K., Mill, J., and Schalkwyk, L.C. (2013). A data-driven approach to preprocessing Illumina 450K methylation array data. BMC Genomics *14*, 1–10. https://doi.org/10.1186/1471-2164-14-293/TABLES/2.
7. Ritchie, M.E., Phipson, B., Wu, D., Hu, Y., Law, C.W., Shi, W., and Smyth, G.K. (2015). limma powers differential expression analyses for RNA-sequencing and microarray studies. Nucleic Acids Res *43*, e47. https://doi.org/10.1093/NAR/GKV007.
8. Ritchie, M.E., Phipson, B., Wu, D., Hu, Y., Law, C.W., Shi, W., and Smyth, G.K. (2015). limma powers differential expression analyses for RNA-sequencing and microarray studies. Nucleic Acids Res *43*, e47–e47. https://doi.org/10.1093/NAR/GKV007.
9. Phipson, B., Maksimovic, J., and Oshlack, A. (2016). missMethyl: an R package for analyzing data from Illumina's HumanMethylation450 platform. Bioinformatics *32*, 286–288. https://doi.org/10.1093/BIOINFORMATICS/BTV560.
10. Kumar, L., and Futschik, M.E. (2007). Mfuzz: a software package for soft clustering of microarray data. Bioinformation *2*, 5–7. https://doi.org/10.6026/97320630002005.
11. Rice, S.J., Brumwell, A., Falk, J., Kehayova, Y.S., Casement, J., Parker, E., Hofer, I.M.J., Shepherd, C., and Loughlin, J. (2022). Genetic risk of osteoarthritis operates during human skeletogenesis. Hum Mol Genet. https://doi.org/10.1093/HMG/DDAC251.
12. Roadmap Epigenomics Consortium, Kundaje, A., Meuleman, W., Ernst, J., Bilenky, M., Yen, A., Heravi-Moussavi, A., Kheradpour, P., Zhang, Z., Wang, J., et al. (2015). Integrative analysis of 111 reference human epigenomes. Nature *518*, 317–329. https://doi.org/10.1038/nature14248.
13. Quinlan, A.R., and Hall, I.M. (2010). BEDTools: a flexible suite of utilities for comparing genomic features. Bioinformatics *26*, 841–842. https://doi.org/10.1093/BIOINFORMATICS/BTQ033.
14. Vorontsov, I.E., Eliseeva, I.A., Zinkevich, A., Nikonov, M., Abramov, S., Boytsov, A., Kamenets, V., Kasianova, A., Kolmykov, S., Yevshin, I.S., et al. (2024). HOCOMOCO in 2024: a rebuild of the curated collection of binding models for human and mouse transcription factors. Nucleic Acids Res *52*, D154–D163. https://doi.org/10.1093/NAR/GKAD1077.
15. Machlab, D., Burger, L., Soneson, C., Rijli, F.M., Schübeler, D., and Stadler, M.B. (2022). monaLisa: an R/Bioconductor package for identifying regulatory motifs. Bioinformatics *38*, 2624–2625. https://doi.org/10.1093/BIOINFORMATICS/BTAC102.
16. Purcell, S., Neale, B., Todd-Brown, K., Thomas, L., Ferreira, M.A.R., Bender, D., Maller, J., Sklar, P., De Bakker, P.I.W., Daly, M.J., et al. (2007). PLINK: a tool set for whole-genome association and population-based linkage analyses. Am J Hum Genet *81*, 559–575. https://doi.org/10.1086/519795.
17. Das, S., Forer, L., Schönherr, S., Sidore, C., Locke, A.E., Kwong, A., Vrieze, S.I., Chew, E.Y., Levy, S., McGue, M., et al. (2016). Next-generation genotype imputation service and methods. Nat Genet *48*, 1284–1287. https://doi.org/10.1038/NG.3656.

18. McCarthy, S., Das, S., Kretzschmar, W., Delaneau, O., Wood, A.R., Teumer, A., Kang, H.M., Fuchsberger, C., Danecek, P., Sharp, K., et al. (2016). A reference panel of 64,976 haplotypes for genotype imputation. Nat Genet *48*, 1279–1283. https://doi.org/10.1038/NG.3643.
19. Li, H. (2011). A statistical framework for SNP calling, mutation discovery, association mapping and population genetical parameter estimation from sequencing data. Bioinformatics *27*, 2987–2993. https://doi.org/10.1093/BIOINFORMATICS/BTR509.
20. Chang, C.C., Chow, C.C., Tellier, L.C.A.M., Vattikuti, S., Purcell, S.M., and Lee, J.J. (2015). Second-generation PLINK: rising to the challenge of larger and richer datasets. Gigascience *4*. https://doi.org/10.1186/S13742-015-0047-8.
